# Inter- and intracellular mitochondrial communication: signaling hubs in aging and age-related diseases

**DOI:** 10.1186/s11658-024-00669-4

**Published:** 2024-12-18

**Authors:** Meng Zhang, Jin Wei, Chang He, Liutao Sui, Chucheng Jiao, Xiaoyan Zhu, Xudong Pan

**Affiliations:** 1 Department of Neurology, The Affiliated Hospital of Qingdao University, Qingdao, 266000 China; 2 Department of Critical Care Medicine, The Affiliated Hospital of Qingdao University, Qingdao, 266000 China

**Keywords:** Mitochondrial communication, Mitochondrial dysfunction, Aging, Age-related diseases, Signaling hubs

## Abstract

Mitochondria are versatile and complex organelles that can continuously communicate and interact with the cellular milieu. Deregulated communication between mitochondria and host cells/organelles has significant consequences and is an underlying factor of many pathophysiological conditions, including the process of aging. During aging, mitochondria lose function, and mitocellular communication pathways break down; mitochondrial dysfunction interacts with mitochondrial dyscommunication, forming a vicious circle. Therefore, strategies to protect mitochondrial function and promote effective communication of mitochondria can increase healthy lifespan and longevity, which might be a new treatment paradigm for age-related disorders. In this review, we comprehensively discuss the signal transduction mechanisms of inter- and intracellular mitochondrial communication, as well as the interactions between mitochondrial communication and the hallmarks of aging. This review emphasizes the indispensable position of inter- and intracellular mitochondrial communication in the aging process of organisms, which is crucial as the cellular signaling hubs. In addition, we also specifically focus on the status of mitochondria-targeted interventions to provide potential therapeutic targets for age-related diseases.

**Graphical Abstract:**

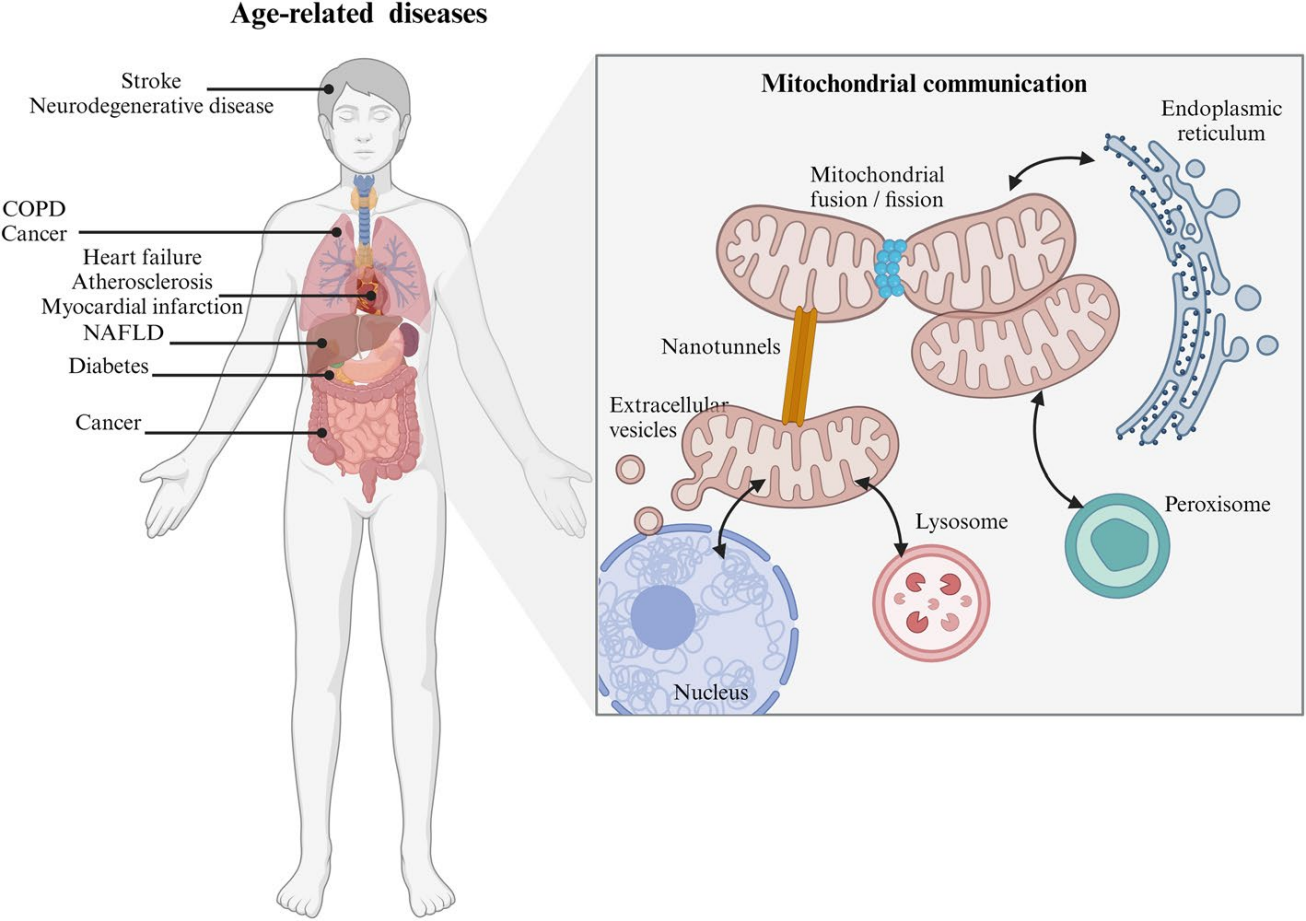

## Introduction

Although commonly called the cell’s powerhouse, mitochondria have roles beyond energy generation. They are essential for pathways within cells and organisms that control immunity, stress reactions, metabolism, and cellular fate [1–3]. To carry out these duties, mitochondria have formed intricate inter- and intracellular communication systems. Within cells, communication pathways consist of direct connections between mitochondria and other subcellular structures and indirect transportation of ions, metabolites, and other intracellular messengers through vesicles. Mitochondria can trigger stress reactions or other cellular alterations that release mitochondrial cytokine factors outside of cells. These factors can move between different tissues and react to immunological challenges originating from outside of cells. Mitochondrial communication refers to the processes by which mitochondria share information and energy capacity with neighboring mitochondria. Additionally, it encompasses the physical interactions and exchange of chemicals and metabolites between mitochondria and other organelles [4]. Nevertheless, the process of mitochondrial communication relies on the synchronized effort of numerous elements, and as a result, it is not infallible. The deregulation of communication between mitochondria and host cells has significant implications and serves as a fundamental element in various pathological diseases, including the aging process.

Aging is an intricate process characterized by a decrease in biological and metabolic functioning, leading to the onset of various age-related illnesses, such as neurological disorders, cardiovascular disorders, metabolic disorders, immune system disorders, and cancer [5]. Over the past 20 years, there has been a significant amount of research on age-related aspects that have provided valuable insights into the aging process. This research has also identified interventions that can potentially extend human lifespan and enhance overall well-being [6–8]. The relationship between cellular and mitochondrial health in the aging process is well recognized as highly interconnected. In addition, it has been shown that affecting the condition of mitochondrial health and communication processes can affect the rate of senescence in a number of experimental organisms. Observations have revealed deficiencies in many molecular components involved in mitochondrial signaling pathways in aging and diseases associated with aging. This suggests that molecular deterioration may occur as a result of disruptions in mitochondrial communication.

However, the precise regulatory mechanism governing mitochondrial communication in aging process is still unknown, which could impede the progress of mitochondria-targeted therapeutics. In this review, we present an up-to-date analysis of the processes and physiological effects of communication in mitochondrial signaling, including the interactions with the hallmarks of aging. In addition, we evaluate the age-related diseases linked to malfunctions in mitochondrial communication. Gaining knowledge about the role of mitochondrial communication in regulating cellular homeostasis will provide a deeper understanding of how impaired mitochondria impact health during disease and the aging process.

## Mitochondrial communication

**Fig. 1:**
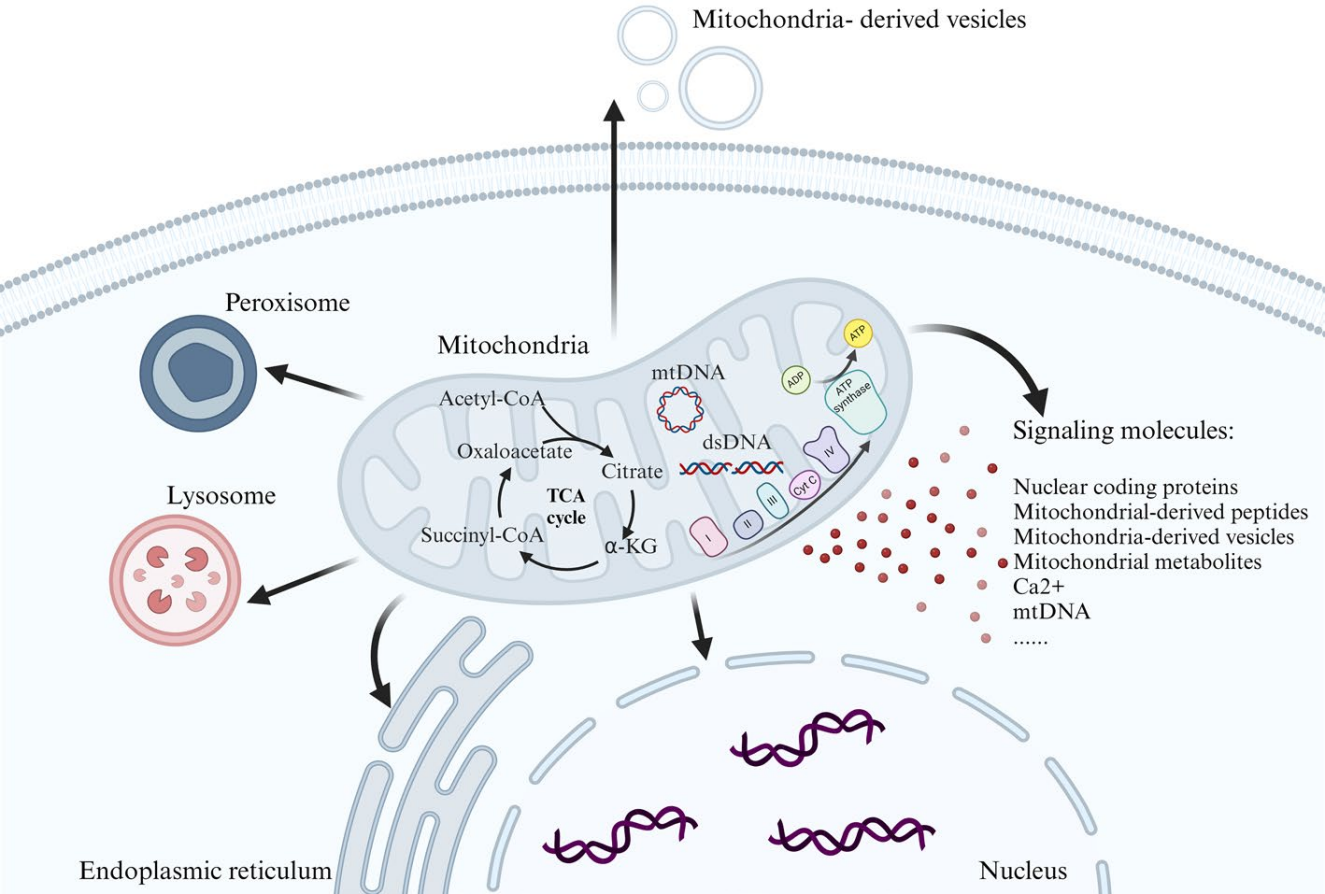
Mitochondrial communication modes The figure was created with Biorender.com

To coordinate cellular processes such as metabolism, stress response, and adaptive nuclear gene expression, mitochondria employ a variety of communication mechanisms. It has been demonstrated that mitochondrial communication is implicated in crucial intracellular and intercellular processes, the nature and extent of which are continually being uncovered. In a dynamic cellular environment, mitochondrial communication transmits signals such as inflammation and metabolic stress to all cells and regions of the body to maintain cellular homeostasis (Fig. 1) [9].

### Intracellular mitochondrial communication

Mitochondria are vital for multiple fundamental biological activities and establish a dynamic functional network to uphold cell homeostasis by interacting with cellular components, including the nucleus, endoplasmic reticulum (ER), peroxisome, lysosome, and other mitochondria. Cells can effectively respond to different environmental changes through the functional network of mitochondrial communication. Disruption in mitochondrial communication can result in the disruption of cell homeostasis and trigger a range of illnesses.

#### Mitoendoplasmic reticulum

In 1959, Copeland et al. discovered for the first time that there were many highly ordered tubular ER structures around mitochondria by transmission electron microscopy [10]. Vance first put forward the concept of mitochondrial-associated endoplasmic reticulum (MAMs) in 1990. MAMs are a lipid raft domain closely related to ER and mitochondria, participating in the control of diverse cellular biological processes and mediating mitochondria and ER [11]. Subsequently, Csordas et al. carried out 3D modeling of transmission electron microscope images, which further confirmed the relationship between the mitochondrial membrane and ER, which is similar but not overlapping, and the distance is between 5 and 25 nm [12]. The communication between mitochondria and ER is accomplished by many protein structures on MAMs. MAMs regulate calcium homeostasis and lipid synthesis and transport, as well as mitochondrial dynamics, mitophagy, and cell apoptosis. The abnormality of quantity, structure or function of MAMs is related to the occurrence of many age-related diseases [13–15].

MAMs are an essential platform for the ER and mitochondrial Ca^2+^ transporter [16]. Many studies have shown that the IP3R–GRP75VDAC complex formed by IP3R, GRP75, and VDAC as classical MAMs proteins is the main structure of Ca^2+^ transport from the ER to mitochondria [17, 18]. Stoica et al. found that ER-resident protein VAPB can bind to mitochondrial protein PTPIP51, affect the interaction between mitochondria and ER, and regulate intracellular Ca^2+^ homeostasis [19]. Studies have shown that the upregulation of Nogo-B protein expression in smooth muscle cells increases the distance between the ER and mitochondria, thereby reducing the calcium transfer from the ER to mitochondria, damaging mitochondrial function and, ultimately, unbalanced the proliferation and apoptosis of smooth muscle cells, thus promoting the occurrence and development of pulmonary arterial hypertension (PAH) [20]. Mfn1/2, which mediates mitochondrial fusion, and Drp1, which mediates mitochondrial division, play essential roles in mitochondrial dynamics [21]. Mfn2 is enriched in the MAMs region and connects the two organelles by forming homologous or heterologous complexes with Mfn1 or Mfn2 on the mitochondrial surface to regulate mitochondrial fusion and mitochondrial uptake of Ca^2+^ [22]. In mouse embryonic fibroblasts, knocking down Mfn2 makes contact between ER and mitochondria closer, resulting in increased Ca^2+^ transfer between the two organelles and increased sensitivity of cells to mitochondrial Ca^2+^ overload [23]. When the mitochondria are divided, the tubular ER is close to the mitochondria, wrapping the contraction sites of the mitochondria and anchoring Drp1 to the mitochondrial membrane to form a Drp1 oligomer contraction ring, which leads to mitochondrial fission [24]. The synthesis of phospholipids mainly relies on the transport of phosphatidic acid between the ER and mitochondrial membranes. PSS1/2 can catalyze the conversion of phosphatidic acid (PA) into phosphatidylserine (PS), which is then transferred to the inner mitochondrial membrane to generate phosphatidylethanolamine (PE) under the action of phosphatidylserine decarboxylase (PSD), CHO-K1. Western blotting results of cells and mouse liver tissue showed that PSS1/2 was highly enriched in MAMs [25]. Studies have shown that the expression of Mfn2 is significantly reduced in liver tissues of patients with nonalcoholic fatty liver disease and mouse models, and specific knockout of Mfn2 from liver cells, the levels of inflammatory factors, and triglycerides in mice increased significantly, promoting the occurrence and development of liver fibrosis and liver cancer. The specific mechanism is that Mfn2 can bind to PS and promote the transfer of PS into the mitochondrial membrane structure, thereby promoting PE synthesis in mitochondria, elucidating the effect of Mfn2 on phospholipid metabolism in maintaining MAMs morphology and function, lipid metabolism, and ER homeostasis [26]. During the process of mitophagy, PINK1, which is localized in the MAMs region, can directly interact with BECN1 to promote the contact between mitochondria and ER and the formation of autophagosome precursor. Silencing PINK1 will reduce the enrichment of BECN1 on MAMs and inhibit the mitophagy process, suggesting that PINK1 is significantly involved in regulating mitophagy [27, 28]. MAMs can not only provide a platform for the occurrence of apoptosis but also recruit apoptosis-related regulatory factors. When the mitochondria divide, the tubular ER is close to the mitochondria and wraps the mitochondria at the fission site. When cells are exposed to apoptosis stress, BAX in the cytoplasm is activated and recruited to the fission site of mitochondria wrapped in ER, which promotes the permeability of mitochondrial outer membrane (MOMP) and the release of cytochrome C, thus inducing apoptosis [24].

#### Mitonucleus

**Fig. 2:**
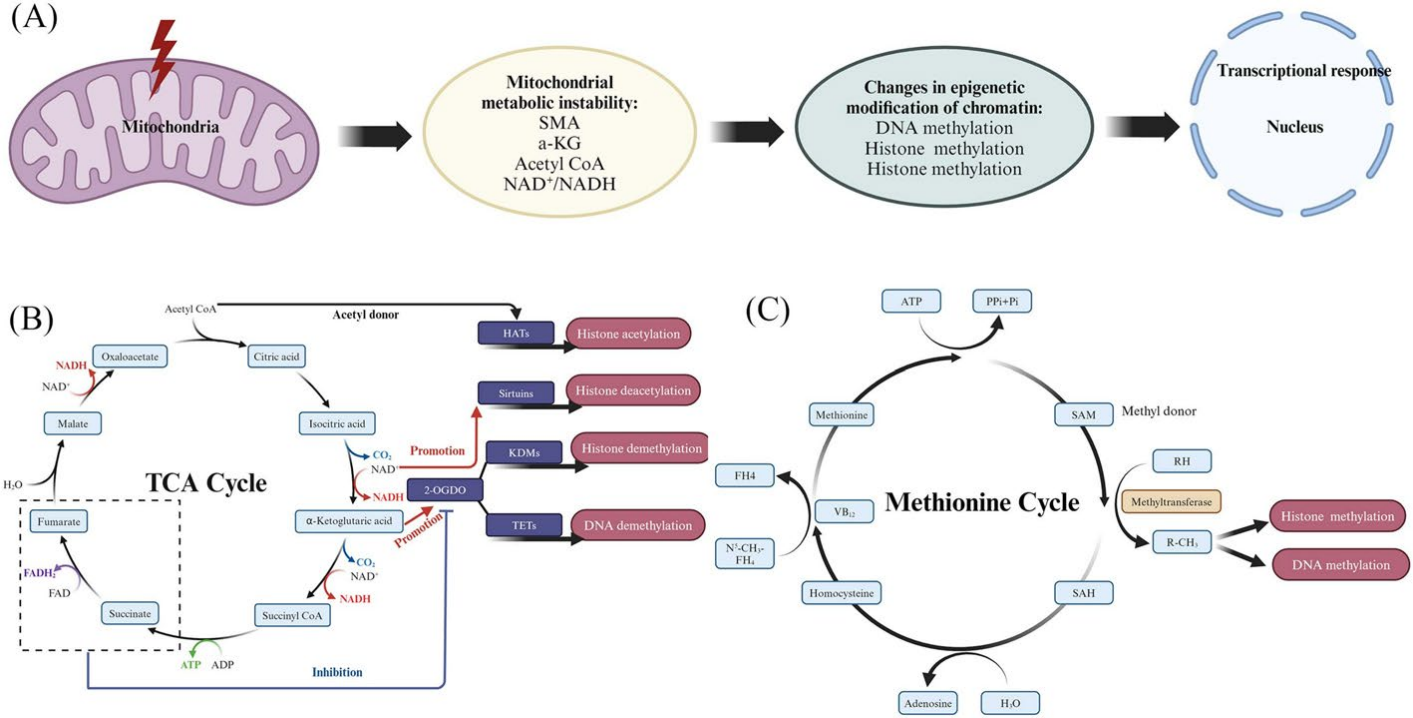
Mitochondrion–nuclear communication and mitochondrial metabolites for epigenetic modifications The figure was created with Biorender.com

Mitochondria serve as the central hub for cellular energy metabolism. However, most of the mitochondrial proteins are encoded by nuclear genes, and only 13 subunits of the electron transport chain complex (ETC) encode genes. Recent studies have confirmed the physiological function of CYTB-187AA, the 14th protein encoded by mitochondrial DNA (mtDNA) [29]. Therefore, mitochondria must communicate with the nucleus continuously to coordinate the expression and assembly of oxidative phosphorylation complexes to maintain mitochondrial function (Fig. 2). The metabolic state of cells not only changes during aging but also responds to environmental stimuli. Stress signals convey the functional status of mitochondria to the nucleus to promote gene transcription that adapts to the environment. Impaired mitochondrial function can lead to mtDNA loss, mtDNA mutation accumulation, respiratory impairment, mitochondrial protein homeostasis and reactive oxygen species (ROS) production. These signals promote communication between mitochondria and nuclei (mitonucleus) to change gene expression, thereby affecting metabolic adaptation and lifespan. Studies have shown that chromatin changes in response to mitochondrial disturbance promote the communication between mitochondria and nuclei and leave epigenetic memories that may affect the aging process [30].

Precise and strict coordination between the mitochondria and the nucleus is essential for maintaining cellular homeostasis. This process happens through two-way communication via anterograde signaling, where the nucleus regulates the expression of mitochondrial genes and makes posttranslational modifications, and retrograde signaling, where the mitochondria regulate the expression of nuclear genes [30, 31]. Mitochondrial function mainly consists of nuclear coding genes to mitochondrial signals, such as PCG1α, nuclear respiratory factor 1 (NRF1), and other coregulatory factors, which regulate the process of mitochondrial biogenesis or enhance mitochondrial activity to meet cellular requirements [32, 33]. On the contrary, in the process of senescence, mitochondrial function decreases due to internal disturbance of mitochondria or accumulation of mtDNA mutations, resulting in decreased OXPHOS activity, changes in tricarboxylic acid (TCA) cycle-related enzymes, increase of ROS, and imbalance of mitochondrial protein homeostasis [34, 35]. This mitochondrial dysfunction will regulate the expression of related nuclear genes, leading to changes in the expression of age-related genes and metabolic reediting. For a long time, the intermediate metabolites of mitochondria have been regarded as byproducts of cell metabolism. However, more and more studies have pointed out that these intermediate metabolites can be used as signals to regulate epigenetic modification in the nucleus and further regulate nuclear gene expression. This affects cell homeostasis and senescence. It includes acetyl-coenzyme A (acetyl-CoA), α-ketoglutaric acid (α-KG), nicotinamide adenine dinucleotide (NAD^+^), and methionine [30, 36, 37]. As potential longevity regulators, they form a dynamic regulatory network with the nucleus, promote cell homeostasis and regulate the expression of age-related metabolic genes, and make cells respond to different metabolic conditions and aging stress.

However, our comprehension of the relationship between mitochondrial–nuclear stress signals remains restricted, despite these advancements. It is crucial to ascertain the impact of mitochondrial metabolites on the regulation of specific sites of epigenetic modifications in a manner that is specific to a particular tissue. Future studies will aid in the development of therapies targeting mitochondrial–nuclear communication to induce advantageous epigenetic modifications that can delay the aging process or alleviate age-related illnesses.

#### Mitolysosome

Lysosomes are mainly responsible for clearing inactivated biological macromolecules or damaged organelles. There is direct physical contact between mitochondria and lysosomes, which differs from the contact formed by lysosomes targeting damaged mitochondria. Mitochondrial–lysosomal (mitolysosome) contact is continuously formed and dissociated in normal cells. Functionally, it allows two-way regulation of mitochondrial and lysosomal dynamics [38]. In the contact formation stage, Ras-associated GTP binding protein 7 (Rab7) promotes contact by binding to GTP on the lysosomal membrane. The member of the Tbc1 domain family 15 (TBC1D15) is recruited to the mitochondria by the mitochondrial fission 1 protein (FIS1). TBC1D15 binds to the RAB7 at the contact site and hydrolyzes guanosine triphosphate (GTP) to guanosine diphosphate (GDP), which drives the hydrolysis of RAB7-GTP, thus relieving contact [39]. Mitochondrial–lysosome contact is significant in cardiac dysfunction induced by acute myocardial infarction. Three days after myocardial infarction in adult rats, mitochondrial–lysosome contact is abnormal, the lysosome is enlarged, and damaged mitochondria can not be cleared. This abnormal function is related to the downregulation of TBC1D15 levels. TBC1D15 relieves mitochondrial–lysosomal contact through the FIS1/Rab7 pathway, and then, it depends on the lysosome for mitochondrial autophagy. Overexpression of TBC1D15 can restore myocardial contractile function and reduce myocardial infarction size and myocardial interstitial fibrosis [40].

#### Mitoperoxisome

Peroxisomes and mitochondria (mitoperoxisome) are linked both physiologically and functionally [41], and effective metabolic intermediate transfer requires this intimate contact. Together, these two organelles facilitate essential biological functions, including peroxisomal growth, lipid metabolism, redox balancing, and antiviral signaling. β-Oxidation in mammals necessitates the presence of both mitochondria and peroxisomes, and there is a requirement for the transfer of metabolites between peroxisomes and mitochondria in both directions. Due to the limited ability of peroxisomes in mammals to metabolize fatty acids with long chains, it is necessary to transport intermediate products to mitochondria in the form of acylcarnitine ester or free acids to assure their complete oxidation. In addition, these coordinated metabolic processes generate ROS, and changes in the metabolism of peroxisomal ROS have an effect on the mitochondrial redox balance [42]. Additionally, to sustain peroxisomal β-oxidation, NADH produced within peroxisomes must be directed to the mitochondria for effective energy regeneration of NADH into NAD^+^ [43]. The SLC25A17 gene encodes peroxisomal membrane protein 34 (PMP34), the only transporter found in human peroxisomes that exhibits substrate selectivity toward NAD^+^ [44]. In the presence of pyrimidine deprivation, peroxisomes-derived ether lipids are responsible for driving the assembly of mitochondrial respiratory supercomplexes, which is an additional metabolic link between peroxisomes and mitochondria [45]. Peroxisomes mainly undergo proliferation through the expansion and asymmetric division of existing organelles. The peroxisomal fission machinery consists of Pex11b, Fis1, MFF, and DRP1. With the exception of Pex11b, these components are also found in mitochondria, indicating a coordinated division process in specific circumstances [46].

It has been observed that peroxisomes and mitochondria are in contact with one another in mammalian cells [47]. However, the components responsible for tethering are still challenging to find or understand. The ACBD2 protein, which is found in both peroxisomes and mitochondria, has been suggested as a biological mechanism that brings peroxisomes and mitochondria closer together, hence enhancing steroid production in Leydig cells [48]. Peroxisomal malfunction is known for triggering mitochondrial abnormalities, demonstrating the significance of peroxisomes for proper mitochondrial activity [49].

### Intercellular mitochondrial communication

Intercellular communication and the transfer of cellular components are crucial for maintaining the balance of multicellular organisms, and the transcellular transport of mitochondria serves as a prominent example of this exchange. In physiological contexts, the process of mitochondrial transfer is associated with biological growth, energy regulation, and removal of detrimental substances, playing crucial roles in preserving the quality of mitochondria. Mitochondria are indispensable for a multitude of vital biological processes, such as oxidative metabolism and biomolecular synthesis, and are particularly susceptible to dysfunction in pathological conditions. Significantly, extensive mitochondrial damage will intensify the deficiencies in the system responsible for maintaining mitochondrial quality. This will stimulate increased transfer of active mitochondria, restore healthy mitochondria from external sources, and eliminate damaged mitochondria from within the body to promote disease outcomes [50].

#### Intercellular mitochondrial transfer

For a considerable period of time, it has been widely believed that mitochondria persist within cells throughout an organism’s lifespan and are passed down only from the mother [51]. However, under some circumstances, mitochondria have the ability to be released from the cell and moved across different cells [52]. Various cell types, such as lymphocytes, neurons, and heart muscle cells, can transfer or receive mitochondria from one another [53]. Organelle exchange is a special form of intercellular communication, which allows one-way or two-way transport of small molecules or ions and intracellular structures, including mitochondria, lysosomes, endosomal vesicles, and plasma membrane modules [54]. Research has indicated that the transmission of mitochondria between cells serves as a protective mechanism, preventing mitochondrial dysfunction that may occur in stressed cells [55].

There are many methods to replace damaged mitochondria in recipient cells. Coincubation is the simplest method, and the transfer efficiency differs in different cell lines. Microinjection and other invasive techniques, such as nanomaterials, can also inject mitochondria into human cells and quickly replace their endogenous intrinsic mtDNA but with lower efficiency than coincubation methods [56, 57]. To facilitate mitochondrial internalization into recipient cells, other techniques have been developed, such as binding mitochondria to the cell-penetrating peptide Pep-1, labeling mitochondria with magnetic beads of mitochondrial outer membrane translocation enzymes, and increasing mitochondrial uptake by MitoCeption [58]. A number of age-related disorders, including Parkinson’s disease (PD), stroke, diabetes, and metabolic syndrome, have shown that mitochondrial activity in damaged cells can be preserved through endogenous or exogenous mitochondrial transfer. This has been demonstrated in a string of cases, highlighting the effectiveness of mitochondrial transfer as a new model of regenerative therapy in aging [59]. Because the effectiveness of mitochondrial internalization into diseased tissues depends on the number and quality of mitochondrial organelles and their appropriate mode of transport, the efficacy of mitochondrial therapy may vary between patients. If mitochondrial transfer is to be applied to the clinic, a better understanding of the mechanisms of mitochondrial transfer and cellular uptake is needed.

#### Mechanisms of mitochondrial transfer

**Fig. 3:**
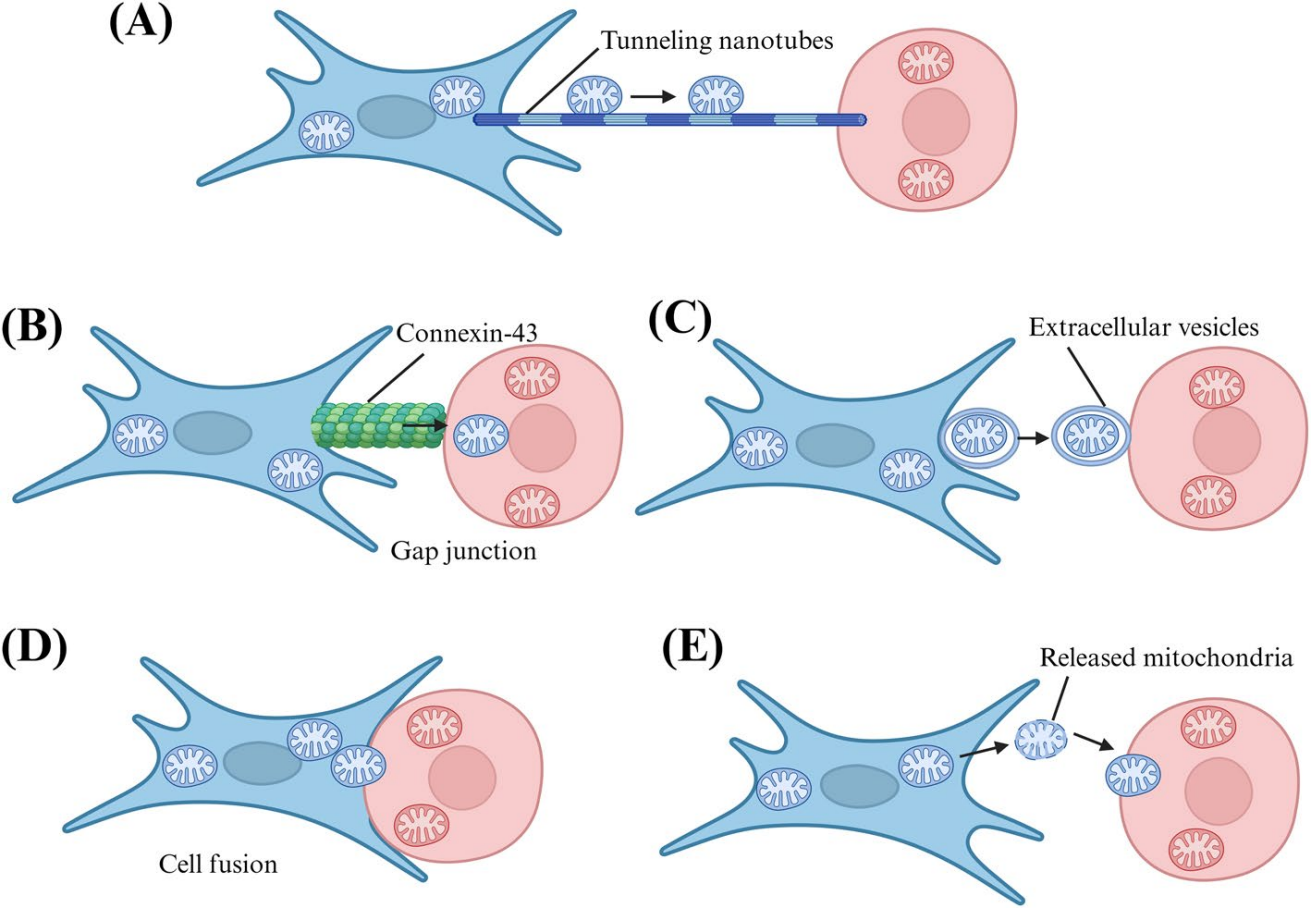
Mechanisms of mitochondrial transfer. **A** Tunneling-nanotubes-mediated mitochondrial transfer. **B** Gap-junction-mediated mitochondrial transfer. **C** Microvesicle-mediated mitochondrial transfer. **D** Cell-fusion-mediated mitochondrial transfer. **E** Endocytosis-mediated mitochondrial transfer The figure was created with Biorender.com

Mechanisms of mediating intercellular mitochondrial transfer include the formation of intercellular tunnelling nanotubes (TNT) or extracellular vesicles (EVs), connexin-43 (Cx43), cell fusion, and mitochondrial extrusion (Fig. 3) [50, 60, 61]. TNT and Cx43 maintain communication between the two connected cells through the membrane tube structure, while EV allows information transfer between the two separated cells, ensuring long-distance communication [62]. In fact, intercellular mitochondrial transfer provides a new mode of intercellular signaling that can occur in vivo and function under a number of unhealthy circumstances to restore damaged cell function [63].

In in vitro and in vivo experiments, TNT is formed between different cell types and promotes the selective exchange of organelles, membrane vesicles, small soluble cytoplasm, and membrane molecules. The establishment of nanotubes begins with the formation of a membrane-like process that shrinks upon arrival at the recipient cell, leaving an ultrafine structure separated from the substrate. TNT is essential for effective mitochondrial transfer, and if the formation of TNT is inhibited by chemical inhibitors or mechanical stress, the mitochondrial exchange can be reduced [64]. Mitochondrial transfer via TNT is usually unidirectional, from the cell initiating TNT formation to the recipient cell [65]. However, there have been a few reports of two-way transfers [66]. Mitochondrial damage is the main trigger of mitochondrial transfer based on TNT. Complete loss of mitochondrial function (including mtDNA depletion or addition of mitochondrial inhibitors) activates mitochondrial transfer. During ischemia–reperfusion injury, mesenchymal stem cells (MSCs) transport mitochondria to damaged endothelial cells using TNT-like structures. This transfer helps prevent endothelial cell death by restoring normal aerobic respiration [67]. Similarly, MSCs can transfer mitochondria to myocardial cells after I/R injury through TNT structure and improve cell survival [68]. In a model of *Escherichia coli* pneumonia, mitochondrial transfer between TNT MSCs and innate immune cells enhances the ability of alveolar macrophages to phagocytic invading bacteria [69]. Other favorable conditions for TNT formation are the induction and stimulation of P53 and nanotube formation in hippocampal astrocytes and neurons by serum starvation or hydrogen peroxide [70]. Similarly, hyperglycemic or acidified media and cytokines that stimulate epithelial–mesenchymal transformation increase mitochondrial transfer through TNT formation [71]. EV is a membrane-containing vesicle shed by cells, which contains protein, lipids, and nucleotides, and plays an important role in intercellular communication. According to its origin, size, and molecular composition, EV can be divided into microbubbles, exosomes, and apoptotic bodies. EV is a tool of intercellular communication, which exists in many physiological and pathological processes and can be used as a biomarker of health and disease [72]. Mitochondrial proteins and mtDNA loaded in different EVS are not clear, but mitochondrial components have been detected in them. Larger EVs can contain complete mitochondrial particles and mtDNA, which can be seen in MSC and participate in its intercellular mitochondrial transfer. It is found that MSC can shed EV, including exosomes (50–100 nm in diameter) and vesicles (0.1–1 mm in diameter), and enter the extracellular space for mitochondrial phagocytosis and delivery of microRNA (miRNA) [73]. Up to now, the mechanism of cell-to-cell transfer of free mtDNA across the mitochondrial inner membrane and outer membrane is still unclear, and in the process of cell-to-cell mitochondrial transfer, EV is most likely to mediate the transfer of the whole mitochondrial particle and restore mitochondrial function. Cell fusion is a process in which two independent cells share organelles and cytoplasmic compounds through the fusion cell membrane. Permanent cell fusion allows cells to share cytoplasm and have unique karyotypes. In contrast, some cell fusion allows short and direct intercellular communication and exchange of a variety of protein complexes and organelles (including mitochondria). It is reported that mature stem cells and embryonic stem cells can fuse with cardiomyocytes, hepatocytes, and neurons, helping to maintain cell differentiation and plasticity [74]. Injury and inflammation can promote cell fusion of target organs, and myeloid cells and lymphocytes can fuse with different tissues in the event of injury or inflammation [75]. When stem cells are used to treat myocardial infarction, partial or whole cell fusion can occur between stem cells and cardiomyocytes, restoring mitochondrial function and promoting cardiomyocyte regeneration [76]. Human adipose stem cells were cocultured with mouse cardiomyocytes, and it was found that F-actin connections were formed between the cells, indicating that mitochondria can participate in the process of cell function recovery through partial cell fusion [77]. When MSC was cocultured with skin fibroblasts from patients with mitochondrial diseases, it was observed that the abnormal mitochondrial morphology of skin fibroblasts was saved from the fission state to the fusion state, and mitochondrial function was restored [78]. Connexins form gap junctions after oligomerization, allowing cells to connect and transfer small molecular cellular components. Cx43 plays an important role in regulating intercellular mitochondrial transfer. In a Ca^2+^-dependent manner, connexins regulate intercellular mitochondrial transfer from MSC to alveolar epithelial cells damaged by lipopolysaccharide through the formation of TNT and EV to restore alveolar biological energy, thus protecting acute lung injury [79]. In addition, the gap junction can connect the mesosome particles to the plasma membrane to form a channel, and the small molecules can spread into the damaged neurons through the connexin half channel composed of Cx43 [80]. Mitochondrial squeeze is another mechanism of mitochondrial transfer, which can release mitochondria or mitochondrial components in cells under certain conditions. For example, when a large amount of ROS is produced, HeLa cells can squeeze and release mitochondrial fragments [81]. Mitochondrial compression occurs not only in vitro but also in vivo, such as platelets squeeze functional mitochondria wrapped in particles and free organelles, thus enhancing the inflammatory response [82]. In addition, when mouse hepatocytes were treated with an anti-FAST antibody, mitochondria were detected in perisinus space and serum, indicating that mitochondrial compression occurred [83].

## The mediators of mitochondrial communication

### Nuclear coding proteins

The regulation of communication between mitochondria and nuclei is very complex, which can be simply summarized as forward regulation from nucleus to mitochondria and reverse regulation from mitochondria to nucleus [30]. In forward regulation, different stimuli regulate mitochondrial function by producing nuclear transcription factors to ensure the normal expression of proteins in mitochondria and the normal progress of various physiological functions. Forward regulation completes the instruction transmission from the nucleus to mitochondria through a variety of signal molecules, regulates mitochondrial biogenesis and mitochondrial function, and responds to different environmental stimuli. The loss of forward regulation will lead to the destruction of mitochondrial homeostasis, leading to various cell dysfunction and age-related diseases.

Nuclear respiratory factor 1 (NRF1) activates the expression of mitochondrial transcription factor A (TFAM). TFAM enters the mitochondria and binds to mtDNA and recruits mitochondrial RNA polymerase and mitochondrial transcription factor B2 (TFB2M) at the promoter to start transcription, promoting mitochondrial biogenesis [84–86]. For example, overexpression of NRF1 can activate the transcriptional process of myocardial regeneration and induce the regeneration of damaged cardiac myocytes in newborn mice. Overexpression of NRF1 in adult mouse hearts can reduce myocardial ischemia–reperfusion injury (MIRI) [61].

Nuclear factor erythroid 2-related factor 2 (NRF2) is a multifunctional transcription factor regulating antioxidant stress, which can bind to Kelch-like epichlorohydrin-related protein 1 (Keap1). Under steady-state conditions, Keap1 binds to NRF2 to cause its ubiquitin degradation and inhibit the activity of NRF2. In the stress response, Keap1 activity decreases or binding to NRF2 decreases, and NRF2 escapes ubiquitin and accumulates in cells. After the accumulated NRF2 is transported to the nucleus, it activates the antioxidant responsive element (ARE) and promotes the antioxidant process [87]. Keap1/NRF2 is an important antioxidant signal pathway in the human body. Targeting the Keap1/NRF2 pathway can improve the symptoms of many cardiovascular diseases. For example, phosphoglycerate mutase family 5 reduces ROS-induced oxidative stress and ferroptosis in mice with heart failure through Keap1/Nrf2 signal pathway [88]. Targeting the Keap1/NRF2 signal pathway can reduce mitochondrial apoptosis and oxidative stress, which can improve the therapeutic effect of lung injury [89].

The activities of various transcription factors are affected by transcription coactivators, and the most studied one is peroxisome proliferator-activated receptor-γ-coactivator 1α (PGC-1α), which regulates mitochondrial energy metabolism by regulating the activity of transcription factors [30]. In the state of exercise, excessive ATP consumption, decreased ATP level or increased AMP/ATP ratio will activate the energy receptor AMPK [adenosine-5′-monophosphate (AMP)-activated protein kinase], activation of AMPK increases the level of PGC-1α [90], and PGC-1α activates its downstream NRF1 and NRF2 nuclear genes, thereby promoting mitochondrial biosynthesis and energy metabolism to meet the energy consumption during exercise [91]. In vascular smooth muscle cells, PGC-1α regulates small noncoding RNA miR-378a by binding to NRF1, and the PGC1α/NRF1/miR-378a axis can protect blood vessels from smooth muscle cell proliferation, migration, and inflammation induced by free fatty acids [92]. The high expression of miR-378a has the function of preventing atherosclerosis, and PGC-1α in human aortic endothelial cells incubated with oxidized low-density lipoprotein is significantly decreased [93], resulting in the inhibition of miR-378a, which is a possible cause of atherosclerosis.

### Mitochondrial-derived peptides

Mitochondrial-derived peptides (MDP) is a new peptide, which is a mitochondrial genome encoded by a small open reading frame. Although MDP can be detected in a variety of tissues and plasma, the mechanism of its secretion and release is still unclear. It has been found that it can regulate mitochondrial bioenergetics and mitochondrial metabolism and has a variety of biological effects, such as helping to maintain mitochondrial function and cell viability under stress, giving full play to cytoprotection, improving metabolic markers and so on. In recent years, MDP has been found in vascular endothelium, cardiomyocytes and islet cells, and its therapeutic value in glucose and lipid metabolic diseases, cardiovascular diseases, Alzheimer’s disease (AD), and other diseases has been proved and affirmed [34, 94–96].

Humanin (HN) is the first member of the MDP family and the most studied peptide in the family. HN is an autopsy of patients with AD performed by Japanese scholar Hashimoto in 2001. A copy DNA library containing an open reading frame was extracted from the undamaged brain region of the occipital cortex. A linear peptide encoding 24 amino acids was found after functional testing. HN can effectively inhibit a variety of familial AD gene mutations and β-amyloid protein-induced neuronal apoptosis, which is considered to be a specific neuroprotective peptide for AD [97]. Cell surface receptors that can activate HN signaling pathways have been identified, including signal transducers and activators of transcription and extracellular regulated protein kinases (ERK) 1 and 2, which are related to cell proliferation and survival [98]. With the deepening of research, it has been found that HN exists in the pathological process of diabetes, myocardial ischemia, atherosclerosis, and other diseases [99–101]. It is secreted by cellular stress and has extensive cytoprotective and neuroprotective effects in these diseases. Now, studies have confirmed that it has the functions of anti-apoptosis, reducing inflammatory reactions and antioxidative stress and improving insulin sensitivity [102–105]. MOST-c is a linear peptide that encodes 16 amino acids in the open reading frame of mitochondrial 12SrRNA. It is a newly found bioactive MDP encoded in mtDNA. The main target of MOST-c is skeletal muscle, which is considered the first peptide in MDP that regulates gene expression in the nucleus by interacting with transcription factors, producing retrograde signal molecules. In the nucleus, MOST-c interacts with antioxidant regulatory transcription factors, such as transcription factor 1 and transcription factor 2, to stimulate the transcription of target genes involved in mitochondrial protection. The cytoprotective effect of MOST-c may be related to this new mechanism. Modern studies have proved that MOST-c can improve muscle metabolism, increase insulin sensitivity, and regulate fat metabolism by increasing glucose utilization and fatty acid oxidation and changing mitochondrial function and nucleotide metabolism [106–110]. SHLP1–6 is another of six small HN-like peptides found in HN’s 16SrRNA gene, all of which are encoded by a small open reading frame. Studies have found that a single SHLP exists in the kidney, spleen, heart, brain, and other organs, with different biological effects. SHLP is a bioactive peptide that regulates cell function. At present, related studies are mainly focused on SHLP2 and SHLP3, which have similar cytoprotective effects to HN and have effects on apoptosis and metabolism [94, 95]. For example, SHLP2 has the effect of antioxidant stress, which can improve insulin sensitivity in the central and peripheral system [94]. The level of SHLP2 in the cycle decreased with age. In addition, similar to HN, SHLP2, and SHLP3 can induce phosphorylation of ERK and activator of transcription 3 [111].

Studies have found that MDP affects the process of cell aging by regulating the transformation of senescence-related secretory phenotype (SASP), inhibiting oxidative stress and antiapoptosis of aging cells, and interferes with the outcome of age-related diseases [112]. Cell senescence is accompanied by the production of SASP, which can continuously block the progress of the cell cycle and induce adjacent cell senescence or carcinogenesis by paracrine. When senescent cells are not removed by the immune system, SASP will cause chronic inflammation, change tissue structure and function, and, eventually, enter an irreversible aging state of stagnation of growth and proliferation [113, 114]. Some studies have found that the bioenergetics of senescent cells play a key role in the expression of some specific SASP, and MDP plays a cytoprotective role in age-related diseases by regulating mitochondrial energy metabolism and then affecting the transformation of SASP in the process of cell aging, so as to reduce the symptoms of aging, reduce the harm caused by aging, and prolong the healthy period [115]. In senescent cells, the levels of HN and MOST-c increased, and MDP moderately increased mitochondrial respiration and a certain part of SASP expression. At the same time, it was found that MDP enhanced energy metabolism but did not cause senescence, indicating that MDP has a therapeutic regulatory effect on mitochondrial energetics and SASP transformation and is a factor to help senescent cells maintain senescence and prevent deterioration and partial SASP expression [112]. Under stress conditions, MDP helps maintain mitochondrial function and cell viability, plays a cytoprotective role, and may become a candidate for various age-related diseases. The effects of MDP on senescent cells are not only related to SASP, but also delay cell senescence by inhibiting oxidative stress, reducing inflammatory response and apoptosis [116]. Further exploration of the specific mechanism and function of MDP will help to clarify the pathogenesis of diseases related to mitochondria and aging, and provide new ideas and targets for the treatment of diseases.

### Mitochondria-derived vesicles

Extracellular vesicles (EVs) are tiny vesicles produced by cells that can transport biologically active substances across cells or organs [117, 118]. Recent data indicate that specific EV subpopulations include a variety of mitochondrial contents. These mitoEVs have the ability to transfer mitochondrial components to target cells, hence influencing their functioning in various contexts [119]. These intracellular vesicles that contain components of mitochondria are known as mitochondrial-derived vesicles (MDVs) [120]. The creation of MDVs has been suggested as the ancient homeostatic mechanism in live cells at the mitochondrial level, occurring under physiological and mild stress situations [121, 122]. One new MDVs biogenesis pathway involves PINK1/Parkin, an E3 ubiquitin protein ligase with a ubiquitin-like domain at the N-terminus but does not require DRP1 [123–126]. Under conditions of mild stress or little damage to the mitochondria, the curvature of the mitochondrial membrane is initiated, which is then followed by the accumulation of PINK1 [93, 127, 128]. Parkin is subsequently enlisted at the outer mitochondrial membrane (OMM), and the MDVs are severed and liberated through an ambiguous process [128, 129]. The participation of DRP1 in the formation of MDVs has been ruled out due to the fact that MDVs can still form even in the absence of DRP1 [128].

The nanoscale vesicles, ranging from ~70 to 150 nm in diameter, are enveloped by either a single or double membrane. These membranes are known as the outer mitochondrial membrane (OMM) and/or the inner mitochondrial membrane (IMM) [130–132]. MDVs are also the particular carriers for mitochondrial nucleic acids [101, 133–136], proteins [133, 137, 138], lipids [121, 139], fragmented mitochondria [140], and/or other mitochondrial components [141, 142]. At present, intracellular MDVs may be distinguished from other intracellular vesicles by utilizing their distinctive markers, such as OMM, IMM, mitochondrial matrix proteins, and mtDNA [143, 144]. Prior research has demonstrated that MDVs are important in intracellular interactions of the parental mitochondria with lysosomes [129, 136], endosomes [136], and peroxisomes [145, 146]. Further investigations have shown the intercellular involvement of MDVs in immune response regulation [147, 148], in eliminating malfunctioning parts of mitochondria [149], and in transporting functional MDVs to target cells that demand more energy for communication [150].

MDVs are recognized as the primary element of the initial secure process in the mitochondrial quality control (MQC) system, and their potential functions are completely distinct from mitochondrial dynamics and mitophagy [151, 152]. In addition, the quantity of MDVs is augmented by mild stress or the initial phase of mitochondrial malfunction [128]. The MQC system has identified two primary categories of MDVs: steady-state MDVs and stress-induced MDVs [153], both of which can be distinguished by their distinct indicators. Translocase of outer mitochondrial membrane 20 (TOMM20), which is a protein located in the OMM, is primarily present in steady-state MDVs [121]. In contrast, pyruvate dehydrogenase (PDH) is mostly present in MDVs that are induced by oxidative stress [123]. Revealing the creation of MDVs and their functional activities will enhance our understanding of the communication that occurs within and between cells related to mitochondria.

### Mitochondrial metabolites

The disorder of mitochondrial energy metabolism leads to abnormal levels of oxidative phosphorylation and respiratory chain intermediate molecules, thus regulating the activity of related epigenetic modifying enzymes, affecting the epigenetic modification status of the genome and changing the expression of related genes. At the initial stage of injury, protective mechanisms such as mitochondrial unfolded protein response (UPR^mt^) are activated to increase the expression of chaperone molecules, proteases and other related genes to maintain mitochondrial homeostasis; with the aggravation of injury, the overactivation of protective mechanisms leads to genomic instability, increases the expression of aging-related genes, and, finally, leads to apoptosis and senescence. Therefore, the abnormal epigenetic regulation of gene expression mediated by mitochondrial metabolic disorders is an important reason for the initiation and progress of the aging phenotype [30].

The TCA cycle is traditionally recognized for generating essential metabolites necessary to ensure cellular proliferation and survival, in addition to generating bioenergetic intermediates that contribute to the ETC [154]. Several of these metabolites also engage in mitochondria-to-nucleus signaling, where they are utilized as secondary messengers, and their levels serve as a direct indicator of mitochondrial health and metabolic condition [155]. Changes in the quantities and accessibility of these metabolites are incorporated into epigenetic regulation techniques that induce transcriptional modifications in response to different stress conditions and physiological states because they frequently function as substrates or regulators of enzymes involved in chromatin remodeling [3]. Two common changes that affect how mitochondrial metabolites work are acetylation and methylation. These are caused by enzymes that respond to changes in acetyl-CoA and α-KG/succinate levels. These changes affect gene expression in the nucleus through DNA methylation and post-translational modifications of histones, which are important parts of the “histone code” [156]. Besides, *S*-adenosine-l-methionine (SAM) is a major donor of methylation, affecting histone methylation, especially H3K4me3. The changes in mitochondrial metabolism affect SAM levels, which in turn affect histone and DNA methylation, profoundly affecting cell homeostasis and body lifespan [30]. Two important metabolites involved in the process of epigenetic control are FAD^+^ and ATP. FAD^+^ serves as a cofactor in the process of histone demethylation. LSD1, also known as lysine-specific demethylase 1, utilizes FAD^+^ as a catalyst to oxidize methylated lysine residues, resulting in the formation of a highly reactive intermediate capable of hydrolytically eliminating methyl groups. ATP serves as an essential energy source for numerous enzyme activities involved in epigenetic modification. Additionally, it acts as a substrate for kinases responsible for phosphorylating histones [30].

The epigenome’s alterations brought about by metabolites, also known as metabolic epigenetics, are linked to the advancement of disease and the reprogramming of gene expression in response to stress and physiological changes [157]. When mtDNA mutations build up, a condition called heteroplasmy, for example, changes in nuclear gene expression are made easier by abnormal metabolite production that leads to specific epigenetic modifications. These modifications, in turn, help control transcription by changing the way chromatin moves [158]. Alterations in the methylation status of genes encoded in the nucleus in response to deficiencies in the copy number of mitochondrial DNA (mtDNA) have been documented in relation to osteosarcoma and breast cancer [159]. These findings emphasize the association between metabolite-induced changes in epigenetic modifications and transcriptional abnormalities associated with pathogenic phenotypes and the evolution of diseases, thereby emphasizing the critical relationship between genetics, mitochondrial retrograde signaling, and metabolites.

### Ca^2+^ homeostasis

Ca^2+^ are widely present and highly adaptable cellular messengers. They have a vital function in orchestrating and regulating many cellular activities, ranging from cell survival to cell death. The expansive nature of Ca^2+^ cascades is influenced by two significant factors: its capacity to interact with multiple effectors and its ability to self-regulate [160]. Within the cell, the coordination of these signaling events is facilitated by a complex system called the Ca^2+^ toolkit, which consists of several organelles and proteins. The ER, mitochondria, nucleus, and plasma membrane, including its channels and transporters, play a crucial role in facilitating the movement of Ca^2+^ from outside the cell to within the cell [161]. The ER stores intracellular Ca^2+^, while the mitochondria act as crucial Ca^2+^ buffers by detecting and regulating intracellular Ca^2+^ levels [162]. Recent research has emphasized the active function of mitochondrial Ca^2+^ in regulating energy production and programmed cell death, both in normal physiological processes and in disease states. The Ca^2+^ signaling toolbox consists of four main divisions: initiation of Ca^2+^ mobilizing signals in response to stimuli, the release of Ca^2+^ from internal stores into the cytoplasm, activation of Ca^2+^ sensitive activities, and transport of Ca^2+^ from the cytoplasm back to internal stores [163].

Maintaining the balance of Ca^2+^ in the mitochondria relies not only on the entry of Ca^2+^ but also on the pace at which it is removed. During a stable condition, the rate at which calcium ions are released from the mitochondria should be the same as the rate at which they are taken in, to preserve a state of equilibrium. The Na^+^–Ca^2+^–Li^+^ Exchanger (NCLX) is responsible for the export of Ca^2+^ from the mitochondria through the IMM, using Na^+^ or Li^+^ as exchange ions. The NCLX transporter is electrogenic, meaning it imports three Na^+^ ions to extrude one Ca^2+^ ion from the mitochondria. The approximate rate of rotation for the above process is 1,000 cycles per second [162]. Entry of Ca^2+^ into the mitochondria impacts its functionality, leading to temporary depolarization of the mitochondrial membrane potential and governing metabolism [164]. The TCA cycle is a significant metabolic system that is controlled by the amounts of calcium ions in the mitochondria. The function of several enzymes in the tricarboxylic acid (TCA) cycle relies on the amounts of calcium ions within the mitochondria. The enzyme PDH connects the metabolic pathways of glycolysis and the TCA by transforming the last product of glycolysis, pyruvate, into acetyl CoA. Ca^2+^ triggers the activation of pyruvate dehydrogenase phosphatase (PDP), which in turn dephosphorylates the E1 subunit of PDH, leading to its activation [165, 166]. Furthermore, elevated amounts of Ca^2+^ can stimulate the function of the TCA cycle enzymes isocitrate dehydrogenase and oxoglutarate dehydrogenase, hence promoting the TCA cycle [167, 168]. Gaining insight into the mechanisms of intracellular mitochondrial Ca^2+^ homeostasis can enhance our understanding of mitochondrial communication and offer valuable insights for the advancement of therapeutic approaches for age-related illnesses.

### Mitogenome stability

MtDNA can participate in inflammatory response by combining with TLR, NLRP, and other receptors. The TLR pathway is triggered by the binding of DAMPs to neutrophils and activates the subsequent inflammatory response through nuclear factor-κB (NF-κB) signal transduction. The NLRP pathway works through NLRP3 inflammatory bodies [169, 170]. Activation of NLRP3 leads to increased expression of caspase-1, which divides and activates IL-1 β and IL-18. In addition, redox-sensitive inflammatory and inflammatory body-mediated pathways can also cooperate to aggravate the inflammatory response: the cGAS–STING DNA pathway is an integral part of the innate immune system [171, 172]. After binding to mtDNA, the cGAS pathway triggers the phosphorylation of interferonregulatoryfactor3 (IRF-3) through chemotaxis of STING protein, and it acts through TANK-binding kinase (TBK). At the same time, the phosphorylation of IRF-3 can induce the production of type I and type III interferons (β and λ1). Persistent inflammatory stimulation can activate circulating immune cells, which in turn may induce inflammatory pathways by activating mtDNA, thus producing systemic reactions. Cytokines, chemokines, nitric oxide, and ROS released by inflammatory cells in circulation can further induce mitochondrial damage, thus forming a vicious circle and strengthening the whole process [173, 174].

## Mitochondrial communication and hallmarks of aging

**Fig. 4:**
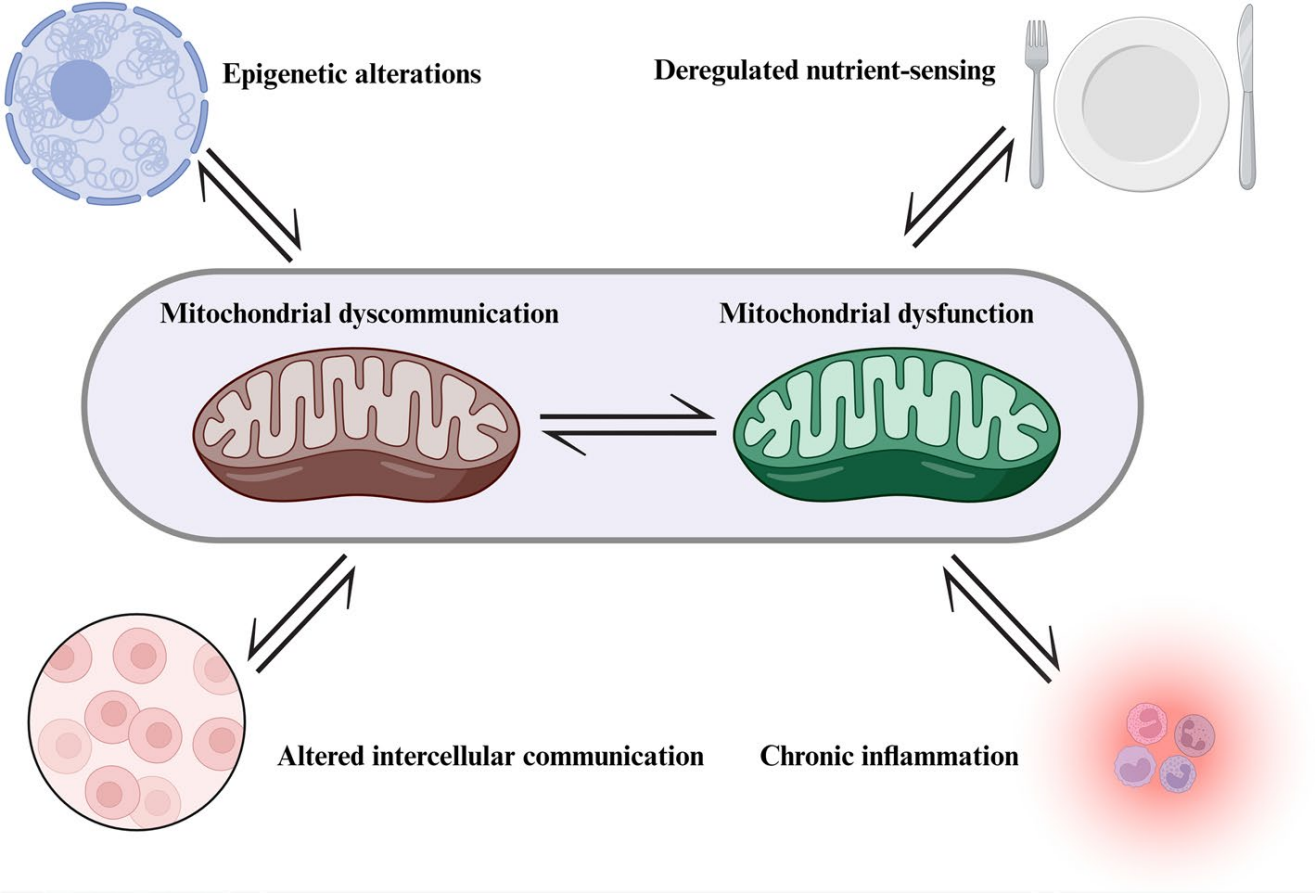
Cross talks between mitochondria communication and hallmarks of aging The figure was created with Biorender.com

There are bidirectional crosstalks between mitochondrial communication and hallmarks of aging. In this review, we focus on mitochondrial dysfunction, epigenetic alterations, chronic inflammation, altered intercellular communication, and deregulated nutrient sensing (Fig. 4).

### Mitochondrial dysfunction

Mitochondria are the center of biological energy and metabolism and widely participate in a variety of biological processes, with complex adaptation mechanisms, which can maintain the normal morphology and function of mitochondria to cope with the effects of mitochondrial damage and aging and other factors. Mitochondrial dysfunction is considered to be one of the hallmarks of aging and is associated with many age-related diseases [175].

Mitochondrial dysfunction refers to the decrease of mitochondrial respiratory capacity and membrane potential. Mitochondria undergo morphological and functional changes with age and continuous exposure to different pressures, which increases the possibility of dysfunction [176]. Similarly, aging is usually accompanied by a decline in the quality and function of mitochondria [108]. The quality control of mitochondria is the basis of maintaining the normal physiological function of mitochondria, and the dynamic balance of mitochondrial production and degradation is an important factor to maintain the function of mitochondria in cells [177]. Mitochondria have a complex quality control system. The mechanism of MQC is multilevel, showing changes at the protein, organelle, and cellular level. As aging occurs, mitochondria with accumulated damage can realize self-repair through a series of mechanisms. There are five types of MQC: UPR^mt^, mitochondrial dynamics, mitophagy, mitochondrial biogenesis, and mitochondrial-derived vesicles [178, 179].

Aging is an intricate process characterized by a decrease in the functionality of several organelles. While mitochondrial malfunction is proposed as a contributing element to the aging process, the precise role of mitochondrial quality control (MQC) in aging remains inadequately comprehended [108]. Increasing data suggest that ROS promote alterations in mitochondrial structure and enhances the buildup of oxidized byproducts via mitochondrial proteases and the mitochondrial UPR^mt^ [180]. MDVs are the primary means of implementing MQC to eliminate oxidized byproducts. In addition, mitophagy assists in the elimination of partially impaired mitochondria to ensure the overall health and functionality of mitochondria. While numerous interventions on MQC have been studied, excessive activation or inhibition of any kind of MQC can potentially worsen aberrant energy metabolism and contribute to senescence caused by mitochondrial malfunction [93, 130]. Therefore, implementing suitable measures to address MQC can potentially slow down the aging process and increase lifetime.

### Epigenetic alterations

The significance of mitochondria in regulating the nuclear epigenome is widely recognized. SAM, a methyl group donor that is universally applicable, is produced in the mitochondria through a metabolic cycle involving one-carbon compounds. Methionine is synthesized in the cytosol by the enzymatic reaction of adenosine-5′-triphosphate (ATP) as a component of the methionine cycle, resulting in the formation of SAM. The methionine cycle in the cytosol is linked to the folate cycle [181]. 5-Methyl tetrahydrofolate (5-MTHF) transfers a methyl group to homocysteine (Hcy), resulting in the formation of tetrahydrofolate (THF) and l-methionine. This process is facilitated by the enzyme methionine synthase (MS), which is dependent on vitamin B12. Additionally, 5-MTHF is involved in the remethylation of Hcy. Therefore, the synthesis of SAM is controlled by ATP generation and folate. The processes of the folate cycle take place in both the mitochondria and cytosol and are connected by the transfer of the serine and glycine amino acid pool via methylenetetrahydrofolate (methionine-THF), which is derived from THF [182]. The mitochondrion is crucial in controlling the transition between nucleotide synthesis and SAM through the mitochondrial bifunctional enzyme (MBE). This enzyme is active in embryonic and cancer cells to facilitate nucleotide synthesis, but it becomes inactive in adult cells to promote the synthesis of SAM [183]. SAM, which is produced in the cytosol, is transferred to the mitochondria through a specialized mitochondrial carrier called SAM carrier (SAMC). Once inside the mitochondria, SAM influences the methylation pattern of the mitochondria [184]. Therefore, any malfunction in the process of one-carbon metabolism would cause changes in the patterns of DNA methylation in mtDNA and disrupt the communication between the nucleus and mitochondria. This communication takes place through the transportation of metabolites from the mitochondria to the nucleus and vice versa, occurring through the cytosol. MiRNAs, which are noncoding tiny RNAs, are observed to be specifically located within the mitochondria [185]. Nuclear-encoded miRNAs regulate nuclear and mtDNA-encoded genes epigenetically and coordinate nuclear–mitochondrial activity. In addition, intermediates of mitochondrial TCA metabolites, such as α-KG, could affect TET protein activity [186], suggesting the potential involvement of mitochondria in the process of demethylation. Noncoding short RNAs have also been identified in the mitochondrial genomes of both mice and humans, although they have received less research attention [187]. The TCA cycle produces metabolites in the mitochondria that regulate epigenetic alterations. These modifications include DNA methylation, histone methylation, and acetylation. The metabolites act as cofactors and facilitate nuclear–mitochondrial communication [30]. Substrates of the TCA cycle, αKG and succinate, perform a vital function in modulating the methylation of DNA and histones as well as the activity of TET demethylase. On the other hand, histone acetylation is regulated by acetyl-CoA [30].

### Chronic inflammation

Not only do mitochondria exchange electrochemical information with the mitochondrial reticulum, but they also exchange mtDNA through membrane contact points between neighboring mitochondria. Nevertheless, when mitochondria experience stress, it can lead to the release of proteins associated with mitochondrial damage (mtDAMPs) into the cytosol, extracellular matrix, and bloodstream [188] and comprise several elements of mitochondria, including mtDNA [189]. One possible method is to distribute material, such as subunits of the oxidative phosphorylation machinery, to other mitochondria. Recently, it has been proposed that mtDNA can not only detect genotoxic stress but also function as a signaling factor to improve the repair of nuclear DNA [190]. Cell-free mtDNA is released into the bloodstream after cellular damage, triggering widespread proinflammatory reactions and activating the immune system [188]. This could be attributed to the resemblance between mtDNA and bacterial DNA, which leads to the immune system identifying mtDNA and triggering a comparable response mechanism employed to combat bacteria [191]. The relationship between mtDNA and the inflammatory response has been demonstrated to occur through the stimulation of polymorphonuclear neutrophils production by mtDNA/Toll-like receptor (TLR)-9 and activation of the NF-κB pathway [192].

Given that inflammation and the alteration of MQC pathways play significant roles in several clinical disorders, such as aging, it is crucial to investigate the involvement of mtDNA in this interplay.

### Altered intercellular communication

The aging process is also characterized by gradual modifications in intercellular communication systems [175]. Intercellular communication can be impacted by mitochondrial dysfunction, which can influence a range of signaling mechanisms such as ROS, mtDNA, the release of MDVs, and metabolite signaling [193]. MDVs can cause the release of fragments of mtDNA, metabolites, and proteins into the space outside of cells. This process triggers immunological signaling pathways, disrupts the secretion of different chemicals, and impedes the transport of functioning mitochondria between cells [193, 194]. These changes have the potential to undermine cellular functioning, impede tissue homeostasis and maybe contribute to the development of age-related disorders such as AD and PD. Gaining a thorough understanding of how mitochondrial malfunction affects communication between cells is essential for appreciating the underlying causes of these disorders and devising effective methods to reduce their impact. Remarkably, the process of mitochondrial transfer can effectively reinstate the proper functioning of mitochondria in the cells that receive them. This has proven to have a significant therapeutic effect in several illness models, such as PD, stroke, and ischemia [194]. Giving mitochondria through a shot into the middle forebrain bundle can lower oxidative damage, stop dopaminergic neurons from dying, and improve movement [195]. However, additional investigation is required to clarify the fundamental processes and investigate potential treatment measures to restore effective communication between cells in the presence of mitochondrial dysfunction.

### Deregulated nutrient-sensing

Mitochondrial dysfunction can disrupt intercellular communication and impair the regulation of nutritional response and energy balance. Aging and metabolic diseases, such as obesity, insulin resistance, and type 2 diabetes, are linked to dysregulated nutrient sensing [175] and can be caused by mitochondrial dysfunction, which leads to compromised energy metabolism, heightened reliance on glucose metabolism rather than fatty acid oxidation, production of ROS, and modified metabolite release and activation of inflammation and stress responses. Mitochondria have crucial functions as sensors of nutrients [196], and there is a strong association between mitochondrial failure and metabolic disorders, including obesity [197]. Mitochondria and ER contact at specific locations known as MAMs. This interaction allows for the exchange of metabolites and calcium. Recent research suggests that MAMs play a crucial role in hepatic insulin signaling and nutrition sensing processes, serving as major hubs for these functions [198]. Furthermore, the primary pathways responsible for sensing nutrients (insulin/IGF1, mTOR, and AMPK) are closely linked to mitochondrial function and mitophagy. There is potential in preserving mitochondrial function, reducing oxidative stress, and restoring metabolic flexibility to address dysregulated nutrient-sensing and develop therapeutic strategies for related metabolic disorders [199].

## Mitochondria-targeted interventions

**Fig. 5:**
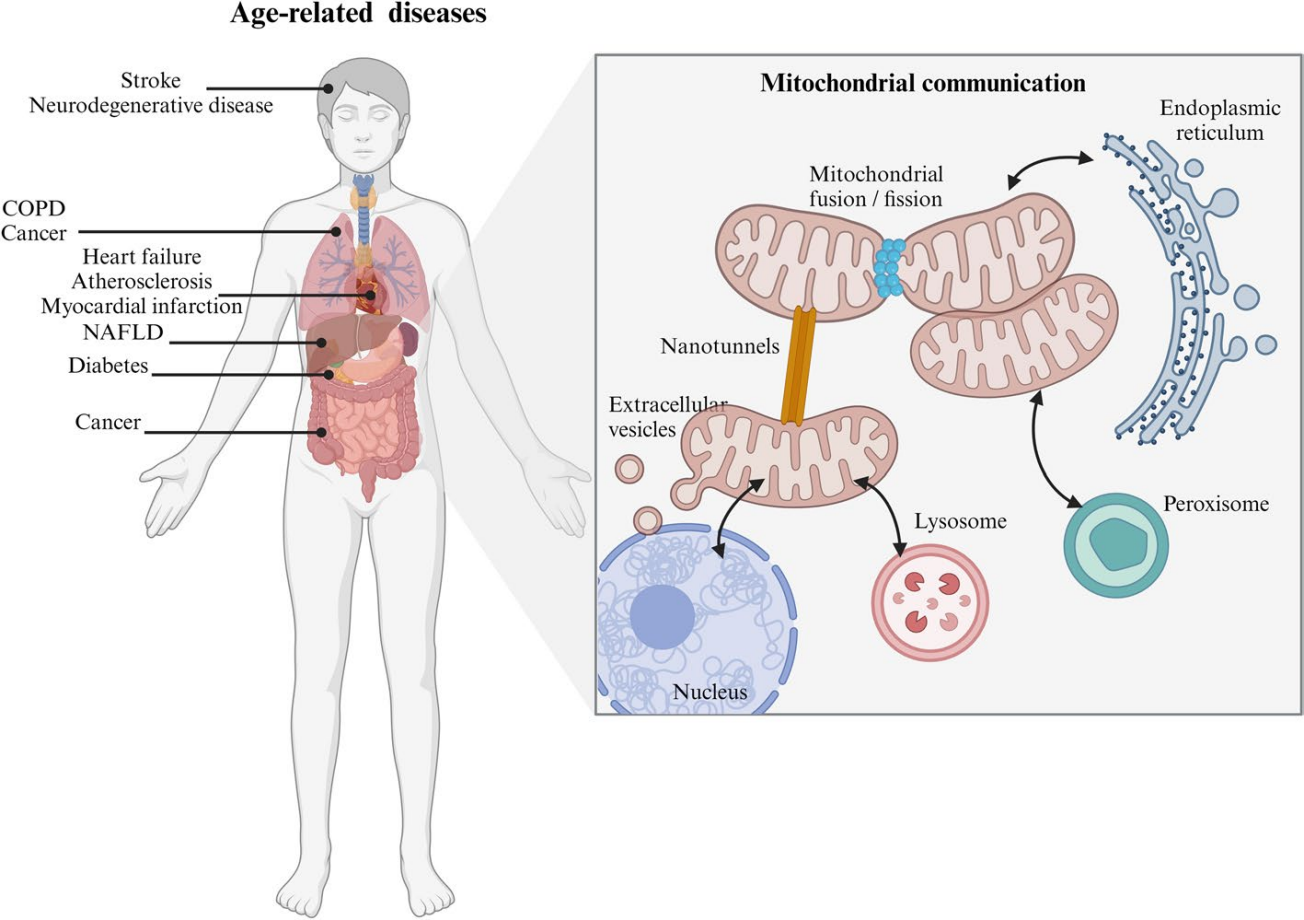
Mitochondrial communication in age-related diseases The figure was created with Biorender.com

Research on therapies that specifically target mitochondria has shown promise in the field of aging and age-related disorders (Table 1 and Fig. 5).

**Table 1:** Mitochondria-targeted interventions in aging and age-related diseases

Target	Interventions	Effect	Age-related diseases	References
Mitochondrial biogenesis	Nicotinamide riboside	Promoting PGC-1α-mediated BACE1 ubiquitination and degradation	Alzheimer’s disease	[200]
	Coq10	Improving lipid metabolism	Obesity/type 2 diabetes	[201]
	Metformin	Activation of AMPK	Diabetes, cardiovascular disease, cognitive decline, and cancer	[202]
	Resveratrol	Elevating Nrf1 and TFAM	Frailty	[203]
Mitochondrial dynamics	Mdivi-1	Inhibition of mitochondrial division	Heart failure and Parkinson’s disease	[204, 205]
	P110	Inhibition of Drp1/Fis1 interaction	Alzheimer’s disease and myocardial infarction	[206, 207]
	SAMβA	Improving metabolism of reactive aldehyde adducts	Heart failure	[208]
	Cilnidipine	Acting as a guanine nucleotide exchange factor for Drp1	I/R heart injury	[209]
Mitophagy	Urolithin A	Activating mitophagy, suppressing NLRP3 inflammasome activation	Alzheimer’s disease, Parkinson’s disease, liver injury, and metabolic cardiomyopathy	[210–213]
	Laempferol and rhapontigenin	Abrogating amyloid-β and tau pathologies	Alzheimer’s disease	[214]
	Oleanolic acid	Modulating of FUNDC1, LC3B, p62, TOM20	Cardiac remodeling	[101]
UPR^mt^	PDI-6	Activation of Wnt/EGL-20	Extending lifespan	[90]
MDVs	Cannabidio	Activation of PINK1/Parkin pathway	Parkinson’s disease	[152]
Antioxidants	Coq10	Enhancing oxidative decomposition of lipids and inhibited de novo synthesis of fatty acids	Obesity/type 2 diabetes	[201]
	MitoQ	Decreasing hydrogen peroxide formation, improving mitochondrial respiration and improving mPTP opening	Alzheimer’s disease, heart failure, and obesity	[215–217]
	MTP-131	Reducing production of reactive oxygen species and cytosolic cytochrome c level	Alzheimer’s disease, heart failure, and myocardial infarction	[218–220]
	*N*-Acetylcysteine	Attenuating lipid peroxidation	Diabetic neuropathy	[221]
	NMN	Ameliorating glucose intolerance by restoring NAD^+^ levels	Type 2 diabetes	[222]
	Resveratrol	Reduced IGF-I levels and increased AMPK and PGC-1alpha activity	Alzheimer’s disease, obesity, and Parkinson’s disease	[223–225]
Membrane potential	FMU200	Reducing oxidative stress and apoptosis	Neurodegenerative disorders	[226]
	Mefunidone	Lowering the ratio of apoptotic cells	Lung fibrosis	[227]
Proteostasis	Nicotinamide riboside triflate	Decreasing Aβ accumulation	Alzheimer’s disease	[228]
Ca^2+^ homeostasis	Ruthenium 360	Protecting against plaque deposition and neuronal death	Alzheimer’s disease	[186]
	CGP37157	Preventing toxic mitochondrial Ca^2+^ overload	Alzheimer’s disease	[229]
Mitogenome stability	Mitochondria-targeted gene delivery	Decreasing the mutated mtDNA ratio by introducing WT mtDNA	Neurodegenerative diseases, diabetes, and cancer	[230]
Mitochondrial transplantation		Supplementing healthy mitochondria	I/R heart injury	[231, 232]

Various approaches have been investigated to selectively transport therapeutic drugs or chemicals to mitochondria, with the goal of improving their performance, reducing oxidative stress, and reinstating cellular homeostasis. These interventions can include several methods, such as tiny chemicals, peptides, antioxidants, and gene therapy. In recent years, researchers have suggested that mitophagy stimulators could be a promising approach to reducing the effects of age-related diseases. NAD^+^ precursors, including nicotinamide riboside, nicotinamide mononucleotide, and similar compounds, have been extensively studied in models of age-related diseases [233]. It has several benefits for mitochondria and recovers the compromised diversity and destroyed microbial compositions in AD [234]. Urolithin A is an additional small molecule that has demonstrated considerable promise in its ability to enhance muscle functions, promote amyloid-β and tau pathology, and stimulate mitophagy [235]. It is noteworthy that peptides designed to enhance mitochondrial functions emphasize elamipretide (SS-31), a synthetic tetrapeptide that interacts with cardiolipin in the inner mitochondrial membrane, which is enriched in mitochondria [236]. Resveratrol, being a potent Sirt1 activator, can stimulate the production of mitochondria and improve oxidative metabolism. Resveratrol not only helps to avoid cardiovascular illnesses but also has a preventive effect on metabolic syndrome and muscular ailments [237]. A recent report introduced a novel strategy involving the utilization of pioglitazone and iron oxide nanoparticles in mesenchymal stem cells to stimulate mitochondrial biosynthesis and enhance the rate of mitochondrial transfer. This approach was successfully applied in a mouse model of pulmonary fibrosis, demonstrating the promising potential of mitochondrial replacement therapy [238]. An alternative approach involves utilizing genome editing techniques to modify mitochondrial genes. For instance, mitochondrially targeted zinc-finger nucleases have been employed in various mouse models [239], mitochondrial-targeted meganucleases (mitoARCUS) [240], double-stranded DNA deaminase-derived cytosine base editor (DdCBE) [241] or an adenine base editor (ABE8e), and a potent AAV9 delivery of RNA-guided Cas9 nuclease [242]. Its primary application has been to rectify severe hereditary genetic disorders [243], but the swift advancement of novel editing technologies may provide comprehensive examinations of age-related alterations in mitochondrial function in preclinical in vivo studies and furnish sufficient proof for potential applications in people. Recently, mitochondrial transmission and transplantation techniques have been utilized for treating mitochondrial illnesses. Additionally, the usage of organelles as therapeutic agents has been tested in animal models and clinical studies, showing promising results. There is potential for increased research, application, and promotion of mitochondrial therapy in the future.

Targeting mitochondria through interventions provides a promising approach to prevent aging and age-related disorders by directly addressing the underlying deficiencies in mitochondria. Although numerous techniques are still in the preliminary phases of research and development, they possess significant potential for enhancing mitochondrial health, safeguarding cellular function, and prolonging a healthy lifespan. Further progress in comprehending mitochondrial biology and enhancing precise intervention approaches could result in revolutionary medicines that significantly influence human health and the aging process.

## Challenges and prospects for the future

Mitochondria, being a major organelle of the cell, have other crucial functions apart from generating energy. Instead of being viewed as a solitary cellular powerhouse, mitochondria should be regarded as a critical signaling hub. The organelle constantly receives information regarding nutrition and health conditions from external sources. In response, it releases molecular messengers to either modify cellular transcriptional, metabolic, or proteomic states or carry out the necessary tasks itself. The active listening and acting tasks performed by mitochondria are most likely a result of their endosymbiotic origin. Originally, mitochondria were independent proteobacterial entities that had their own self-contained cellular functions. However, as they developed, they acquired a necessity and capability to communicate signals with the remaining eukaryotic cells and, subsequently, other tissues within the body.

There are still numerous unresolved inquiries in the domain of mitochondrial communication. The expanding body of research reveals an increasing number of molecular components, organelles, and tissues that are governed by the mitochondrial signal network. However, numerous issues persist: How significantly does mitochondrial communication contribute to the aging process? How is every signal molecule in this huge communication network used by mitochondrial communication? How do we exclude the indirect influence of signal molecules by mitochondria in the process of energy metabolism? Given the complex involvement of mitochondria in so many different signal pathways, how can we target mitochondrial communication and clarify the crosstalk between mitochondrial communication and aging markers to reduce the prevalence of age-related disorders? Much remains to be done to understand the detailed role of mitochondrial communication networks in age-related diseases and how to use this understanding to design treatments.

## Conclusions

Mitochondrial dysfunction can have extensive effects on the entire body, impacting multiple physiological systems and playing a role in the onset of different diseases. Mitochondrial communication is vital in protecting mitochondrial health and safeguarding mitochondrial function by facilitating the transfer of material and information between mitochondria. Still, it is important to remember that increasing mitochondrial communication can also expedite the spread of substances and signals in impaired mitochondria, potentially exacerbating cellular damage. The relationship between cellular health and mitochondrial health in the aging process is well-recognized to be closely interconnected. Furthermore, manipulating the state of mitochondrial health and the exchange of information has been demonstrated to impact the progression of aging in numerous experimental organisms. Observations have revealed defects in many molecular components involved in mitochondrial signaling pathways in aging and age-related illnesses. This suggests that molecular deterioration may occur as a result of disrupted mitochondrial communication. Gaining insight into the process of mitochondrial communication and the interplay between mitochondrial communication and aging would surely enhance our understanding of age-related disorders from a novel standpoint and offer potential targets for their diagnosis and therapy.

## Data Availability

Not applicable.

## Abbreviations

acetyl-CoA: Acetyl-coenzyme A
α-KG: α-Ketoglutaric acid
ARE: Antioxidant responsive element
ATP: Adenosine 5′-triphosphate
DdCBE: Double-stranded DNA deaminase-derived cytosine base editor
ER: Endoplasmic reticulum
ETC: Electron transport chain complex
EVs: Extracellular vesicles
FIS1: Fission 1 protein
GDP: Guanosine diphosphate
GTP: Guanosine triphosphate
Hcy: Homocysteine
IRF-3: Interferonregulatoryfactor3
Keap1: Kelch-like epichlorohydrin-related protein 1
MAMs: Mitochondrial-associated endoplasmic reticulum
MBE: Mitochondrial bifunctional enzyme
methionine-THF: Methylenetetrahydrofolate
MOMP: Mitochondrial outer membrane
miRNA: MicroRNA
MQC: Mitochondrial quality control
MIRI: Myocardial ischemia–reperfusion injury
mitoARCUS: Mitochondrial-targeted meganucleases
MS: Methionine synthase
MSCs: Mesenchymal stem cells
mtDAMPs: Proteins associated with mitochondrial damage
mtDNA: Mitochondrial DNA
5-MTHF: 5-Methyl tetrahydrofolate
NAD^+^: Nicotinamide adenine dinucleotide
NCLX: Na^+^Ca^2+^-Li^+^Exchanger
NF-κB: Nuclear factor-κB
NRF1: Nuclear respiratory factor 1
NRF2: Nuclear factor erythroid 2-related factor 2
OMM: Outer mitochondrial membrane
PA: Phosphatidic acid
PAH: Pulmonary arterial hypertension
PE: Phosphatidylethanolamine
PDH: Pyruvate dehydrogenase
PDP: Pyruvate dehydrogenase phosphatase
PGC-1α: Peroxisome proliferator-activated receptor-γ-coactivator 1α
PMP34: Peroxisomal membrane protein 34
PS: Phosphatidylserine
PSD: Phosphatidylserine decarboxylase
Rab7: Ras-associated GTP binding protein 7
ROS: Reactive oxygen species
SAM: *S*-Adenosine-l-methionine
SAMC: SAM carrier
TBC1D15: Tbc1 domain family 15
TBK: TANK-binding kinase
TCA: Tricarboxylic acid
TFAM: Mitochondrial transcription factor A
TFB2M: Mitochondrial transcription factor B2
THF: Tetrahydrofolate
TLR: Toll-like receptor
TNT: Tunnelling nanotubes
TOMM20: Translocase of outer mitochondrial membrane 20
UPRmt: Mitochondrial unfolded protein response

## References

[1] Monzel AS, Enríquez JA, Picard M. Multifaceted mitochondria: moving mitochondrial science beyond function and dysfunction. Nat Metab. 2023;5(4):546–62. 37100996 10.1038/s42255-023-00783-1 PMC10427836

[2] Spinelli JB, Haigis MC. The multifaceted contributions of mitochondria to cellular metabolism. Nat Cell Biol. 2018;20(7):745–54. 29950572 10.1038/s41556-018-0124-1 PMC6541229

[3] Martínez-Reyes I, Chandel NS. Mitochondrial TCA cycle metabolites control physiology and disease. Nat Commun. 2020;11(1):102. 31900386 10.1038/s41467-019-13668-3 PMC6941980

[4] Boardman NT, Trani G, Scalabrin M, Romanello V, Wüst RCI. Intracellular to interorgan mitochondrial communication in striated muscle in health and disease. Endocr Rev. 2023;44(4):668–92. 36725366 10.1210/endrev/bnad004 PMC10335175

[5] Guo J, Huang X, Dou L, Yan M, Shen T, Tang W, et al. Aging and aging-related diseases: from molecular mechanisms to interventions and treatments. Signal Transduct Target Ther. 2022;7(1):391. 36522308 10.1038/s41392-022-01251-0 PMC9755275

[6] Campisi J, Kapahi P, Lithgow GJ, Melov S, Newman JC, Verdin E. From discoveries in ageing research to therapeutics for healthy ageing. Nature. 2019;571(7764):183–92. 31292558 10.1038/s41586-019-1365-2 PMC7205183

[7] Guarente L, Sinclair DA, Kroemer G. Human trials exploring anti-aging medicines. Cell Metab. 2024;36(2):354–76. 38181790 10.1016/j.cmet.2023.12.007

[8] Salvioli S, Basile MS, Bencivenga L, Carrino S, Conte M, Damanti S, et al. Biomarkers of aging in frailty and age-associated disorders: state of the art and future perspective. Ageing Res Rev. 2023;91: 102044. 37647997 10.1016/j.arr.2023.102044

[9] Son JM, Lee C. Aging: all roads lead to mitochondria. Semin Cell Dev Biol. 2021;116:160–8. 33741252 10.1016/j.semcdb.2021.02.006 PMC9774040

[10] Copeland DE, Dalton AJ. An association between mitochondria and the endoplasmic reticulum in cells of the pseudobranch gland of a teleost. J Biophys Biochem Cytol. 1959;5(3):393–6. 13664679 10.1083/jcb.5.3.393 PMC2224680

[11] Vance JE. Phospholipid synthesis in a membrane fraction associated with mitochondria. J Biol Chem. 1990;265(13):7248–56. 2332429

[12] Csordás G, Renken C, Várnai P, Walter L, Weaver D, Buttle KF, et al. Structural and functional features and significance of the physical linkage between ER and mitochondria. J Cell Biol. 2006;174(7):915–21. 16982799 10.1083/jcb.200604016 PMC2064383

[13] Ding Y, Liu N, Zhang D, Guo L, Shang Q, Liu Y, et al. Mitochondria-associated endoplasmic reticulum membranes as a therapeutic target for cardiovascular diseases. Front Pharmacol. 2024;15:1398381. 38694924 10.3389/fphar.2024.1398381 PMC11061472

[14] He Q, Qu M, Shen T, Su J, Xu Y, Xu C, et al. Control of mitochondria-associated endoplasmic reticulum membranes by protein S-palmitoylation: novel therapeutic targets for neurodegenerative diseases. Ageing Res Rev. 2023;87: 101920. 37004843 10.1016/j.arr.2023.101920

[15] Janikiewicz J, Szymański J, Malinska D, Patalas-Krawczyk P, Michalska B, Duszyński J, et al. Mitochondria-associated membranes in aging and senescence: structure, function, and dynamics. Cell Death Dis. 2018;9(3):332. 29491385 10.1038/s41419-017-0105-5 PMC5832430

[16] Jin C, Kumar P, Gracia-Sancho J, Dufour JF. Calcium transfer between endoplasmic reticulum and mitochondria in liver diseases. FEBS Lett. 2021;595(10):1411–21. 33752262 10.1002/1873-3468.14078

[17] D’Eletto M, Rossin F, Occhigrossi L, Farrace MG, Faccenda D, Desai R, et al. Transglutaminase type 2 regulates ER-mitochondria contact sites by interacting with GRP75. Cell Rep. 2018;25(13):3573-3581.e4. 30590033 10.1016/j.celrep.2018.11.094

[18] Yuan M, Gong M, He J, Xie B, Zhang Z, Meng L, et al. IP3R1/GRP75/VDAC1 complex mediates endoplasmic reticulum stress-mitochondrial oxidative stress in diabetic atrial remodeling. Redox Biol. 2022;52: 102289. 35344886 10.1016/j.redox.2022.102289 PMC8961221

[19] Stoica R, De Vos KJ, Paillusson S, Mueller S, Sancho RM, Lau KF, et al. ER-mitochondria associations are regulated by the VAPB-PTPIP51 interaction and are disrupted by ALS/FTD-associated TDP-43. Nat Commun. 2014;5:3996. 24893131 10.1038/ncomms4996 PMC4046113

[20] Sutendra G, Dromparis P, Wright P, Bonnet S, Haromy A, Hao Z, et al. The role of Nogo and the mitochondria-endoplasmic reticulum unit in pulmonary hypertension. Sci Transl Med. 2011;3(88):88ra55. 21697531 10.1126/scitranslmed.3002194 PMC3744110

[21] Chan DC. Mitochondrial dynamics and its involvement in disease. Annu Rev Pathol. 2020;15:235–59. 31585519 10.1146/annurev-pathmechdis-012419-032711

[22] de Brito OM, Scorrano L. Mitofusin 2 tethers endoplasmic reticulum to mitochondria. Nature. 2008;456(7222):605–10. 19052620 10.1038/nature07534

[23] Filadi R, Greotti E, Turacchio G, Luini A, Pozzan T, Pizzo P. Mitofusin 2 ablation increases endoplasmic reticulum-mitochondria coupling. Proc Natl Acad Sci USA. 2015;112(17):E2174–81. 25870285 10.1073/pnas.1504880112 PMC4418914

[24] Yedida G, Milani M, Cohen GM, Varadarajan S. Apogossypol-mediated reorganisation of the endoplasmic reticulum antagonises mitochondrial fission and apoptosis. Cell Death Dis. 2019;10(7):521. 31285422 10.1038/s41419-019-1759-y PMC6614446

[25] Stone SJ, Vance JE. Phosphatidylserine synthase-1 and -2 are localized to mitochondria-associated membranes. J Biol Chem. 2000;275(44):34534–40. 10938271 10.1074/jbc.M002865200

[26] Hernández-Alvarez MI, Sebastián D, Vives S, Ivanova S, Bartoccioni P, Kakimoto P, et al. Deficient endoplasmic reticulum-mitochondrial phosphatidylserine transfer causes liver disease. Cell. 2019;177(4):881-895.e17. 31051106 10.1016/j.cell.2019.04.010

[27] Barazzuol L, Giamogante F, Brini M, Calì T. PINK1/Parkin mediated mitophagy, Ca(2+) signalling, and ER-mitochondria contacts in Parkinson’s disease. Int J Mol Sci. 2020;21(5):1772. 32150829 10.3390/ijms21051772 PMC7084677

[28] Gelmetti V, De Rosa P, Torosantucci L, Marini ES, Romagnoli A, Di Rienzo M, et al. PINK1 and BECN1 relocalize at mitochondria-associated membranes during mitophagy and promote ER-mitochondria tethering and autophagosome formation. Autophagy. 2017;13(4):654–69. 28368777 10.1080/15548627.2016.1277309 PMC5388214

[29] Hu Z, Yang L, Zhang M, Tang H, Huang Y, Su Y, et al. A novel protein CYTB-187AA encoded by the mitochondrial gene CYTB modulates mammalian early development. Cell Metab. 2024;36(7):1586-1597.e7. 38703762 10.1016/j.cmet.2024.04.012

[30] Zhu D, Li X, Tian Y. Mitochondrial-to-nuclear communication in aging: an epigenetic perspective. Trends Biochem Sci. 2022;47(8):645–59. 35397926 10.1016/j.tibs.2022.03.008

[31] Shukla P, Singh KK. Uncovering mitochondrial determinants of racial disparities in ovarian cancer. Trends Cancer. 2021;7(2):93–7. 33246874 10.1016/j.trecan.2020.10.014 PMC9375692

[32] Dasgupta D, Mahadev Bhat S, Price AL, Delmotte P, Sieck GC. Molecular mechanisms underlying TNFα-induced mitochondrial biogenesis in human airway smooth muscle. Int J Mol Sci. 2023;24(6):5788. 36982859 10.3390/ijms24065788 PMC10055892

[33] Tabebi M, Łysiak M, Dutta RK, Lomazzi S, Turkina MV, Brunaud L, et al. Genetic alterations in mitochondrial DNA are complementary to nuclear DNA mutations in pheochromocytomas. Cancers. 2022;14(2):269. 35053433 10.3390/cancers14020269 PMC8773562

[34] Sturm G, Karan KR, Monzel AS, Santhanam B, Taivassalo T, Bris C, et al. OxPhos defects cause hypermetabolism and reduce lifespan in cells and in patients with mitochondrial diseases. Commun Biol. 2023;6(1):22. 36635485 10.1038/s42003-022-04303-x PMC9837150

[35] Liu Y, Huang Y, Xu C, An P, Luo Y, Jiao L, et al. Mitochondrial dysfunction and therapeutic perspectives in cardiovascular diseases. Int J Mol Sci. 2022;23(24):16053. 36555691 10.3390/ijms232416053 PMC9788331

[36] English J, Son JM, Cardamone MD, Lee C, Perissi V. Decoding the rosetta stone of mitonuclear communication. Pharmacol Res. 2020;161: 105161. 32846213 10.1016/j.phrs.2020.105161 PMC7755734

[37] Wiese M, Bannister AJ. Two genomes, one cell: mitochondrial-nuclear coordination via epigenetic pathways. Mol Metab. 2020;38: 100942. 32217072 10.1016/j.molmet.2020.01.006 PMC7300384

[38] Wong YC, Ysselstein D, Krainc D. Mitochondria-lysosome contacts regulate mitochondrial fission via RAB7 GTP hydrolysis. Nature. 2018;554(7692):382–6. 29364868 10.1038/nature25486 PMC6209448

[39] Onoue K, Jofuku A, Ban-Ishihara R, Ishihara T, Maeda M, Koshiba T, et al. Fis1 acts as a mitochondrial recruitment factor for TBC1D15 that is involved in regulation of mitochondrial morphology. J Cell Sci. 2013;126(Pt 1):176–85. 23077178 10.1242/jcs.111211

[40] Yu W, Sun S, Xu H, Li C, Ren J, Zhang Y. TBC1D15/RAB7-regulated mitochondria-lysosome interaction confers cardioprotection against acute myocardial infarction-induced cardiac injury. Theranostics. 2020;10(24):11244–63. 33042281 10.7150/thno.46883 PMC7532681

[41] Fransen M, Lismont C, Walton P. The peroxisome-mitochondria connection: how and why? Int J Mol Sci. 2017;18(6):1126. 28538669 10.3390/ijms18061126 PMC5485950

[42] Lismont C, Nordgren M, Van Veldhoven PP, Fransen M. Redox interplay between mitochondria and peroxisomes. Front Cell Dev Biol. 2015;3:35. 26075204 10.3389/fcell.2015.00035 PMC4444963

[43] Wanders RJ, Waterham HR, Ferdinandusse S. Metabolic interplay between peroxisomes and other subcellular organelles including mitochondria and the endoplasmic reticulum. Front Cell Dev Biol. 2015;3:83. 26858947 10.3389/fcell.2015.00083 PMC4729952

[44] Agrimi G, Russo A, Scarcia P, Palmieri F. The human gene SLC25A17 encodes a peroxisomal transporter of coenzyme A, FAD and NAD+. Biochem J. 2012;443(1):241–7. 22185573 10.1042/BJ20111420

[45] Bennett CF, O’Malley KE, Perry EA, Balsa E, Latorre-Muro P, Riley CL, et al. Peroxisomal-derived ether phospholipids link nucleotides to respirasome assembly. Nat Chem Biol. 2021;17(6):703–10. 33723432 10.1038/s41589-021-00772-z PMC8159895

[46] Schrader M, Costello JL, Godinho LF, Azadi AS, Islinger M. Proliferation and fission of peroxisomes—an update. Biochem Biophys Acta. 2016;1863(5):971–83. 26409486 10.1016/j.bbamcr.2015.09.024

[47] Vallese F, Catoni C, Cieri D, Barazzuol L, Ramirez O, Calore V, et al. An expanded palette of improved SPLICS reporters detects multiple organelle contacts in vitro and in vivo. Nat Commun. 2020;11(1):6069. 33247103 10.1038/s41467-020-19892-6 PMC7699637

[48] Fan J, Li X, Issop L, Culty M, Papadopoulos V. ACBD2/ECI2-mediated peroxisome-mitochondria interactions in Leydig cell steroid biosynthesis. Mol Endocrinol. 2016;30(7):763–82. 27167610 10.1210/me.2016-1008 PMC5426581

[49] Peeters A, Shinde AB, Dirkx R, Smet J, De Bock K, Espeel M, et al. Mitochondria in peroxisome-deficient hepatocytes exhibit impaired respiration, depleted DNA, and PGC-1α independent proliferation. Biochem Biophys Acta. 2015;1853(2):285–98. 25450972 10.1016/j.bbamcr.2014.11.017

[50] Liu Y, Fu T, Li G, Li B, Luo G, Li N, et al. Mitochondrial transfer between cell crosstalk—an emerging role in mitochondrial quality control. Ageing Res Rev. 2023;91: 102038. 37625463 10.1016/j.arr.2023.102038

[51] Sato M, Sato K. Maternal inheritance of mitochondrial DNA: degradation of paternal mitochondria by allogeneic organelle autophagy, allophagy. Autophagy. 2012;8(3):424–5. 22302002 10.4161/auto.19243

[52] Tan AS, Baty JW, Dong LF, Bezawork-Geleta A, Endaya B, Goodwin J, et al. Mitochondrial genome acquisition restores respiratory function and tumorigenic potential of cancer cells without mitochondrial DNA. Cell Metab. 2015;21(1):81–94. 25565207 10.1016/j.cmet.2014.12.003

[53] Davis DM, Sowinski S. Membrane nanotubes: dynamic long-distance connections between animal cells. Nat Rev Mol Cell Biol. 2008;9(6):431–6. 18431401 10.1038/nrm2399

[54] Rogers RS, Bhattacharya J. When cells become organelle donors. Physiology. 2013;28(6):414–22. 24186936 10.1152/physiol.00032.2013

[55] Jiang D, Gao F, Zhang Y, Wong DS, Li Q, Tse HF, et al. Mitochondrial transfer of mesenchymal stem cells effectively protects corneal epithelial cells from mitochondrial damage. Cell Death Dis. 2016;7(11): e2467. 27831562 10.1038/cddis.2016.358 PMC5260876

[56] Kitani T, Kami D, Matoba S, Gojo S. Internalization of isolated functional mitochondria: involvement of macropinocytosis. J Cell Mol Med. 2014;18(8):1694–703. 24912369 10.1111/jcmm.12316 PMC4190914

[57] Sun C, Liu X, Wang B, Wang Z, Liu Y, Di C, et al. Endocytosis-mediated mitochondrial transplantation: transferring normal human astrocytic mitochondria into glioma cells rescues aerobic respiration and enhances radiosensitivity. Theranostics. 2019;9(12):3595–607. 31281500 10.7150/thno.33100 PMC6587163

[58] Cabrera F, Castañeda V, Morales E, Velarde F, Ortega M, Leon-Sosa A, et al. Early evidence of the artificial transfer/transplant of mitochondria to oocytes and zygotes by MitoCeption. Mitochondrion. 2022;65:102–12. 35618256 10.1016/j.mito.2022.05.006

[59] Liu Z, Sun Y, Qi Z, Cao L, Ding S. Mitochondrial transfer/transplantation: an emerging therapeutic approach for multiple diseases. Cell Biosci. 2022;12(1):66. 35590379 10.1186/s13578-022-00805-7 PMC9121600

[60] Geng Z, Guan S, Wang S, Yu Z, Liu T, Du S, et al. Intercellular mitochondrial transfer in the brain, a new perspective for targeted treatment of central nervous system diseases. CNS Neurosci Ther. 2023;29(11):3121–35. 37424172 10.1111/cns.14344 PMC10580346

[61] Cui M, Atmanli A, Morales MG, Tan W, Chen K, Xiao X, et al. Nrf1 promotes heart regeneration and repair by regulating proteostasis and redox balance. Nat Commun. 2021;12(1):5270. 34489413 10.1038/s41467-021-25653-w PMC8421386

[62] Ribeiro-Rodrigues TM, Martins-Marques T, Morel S, Kwak BR, Girão H. Role of connexin 43 in different forms of intercellular communication—gap junctions, extracellular vesicles and tunnelling nanotubes. J Cell Sci. 2017;130(21):3619–30. 29025971 10.1242/jcs.200667

[63] Liu F, Lu J, Manaenko A, Tang J, Hu Q. Mitochondria in ischemic stroke: new insight and implications. Aging Dis. 2018;9(5):924–37. 30271667 10.14336/AD.2017.1126 PMC6147588

[64] Bukoreshtliev NV, Wang X, Hodneland E, Gurke S, Barroso JF, Gerdes HH. Selective block of tunneling nanotube (TNT) formation inhibits intercellular organelle transfer between PC12 cells. FEBS Lett. 2009;583(9):1481–8. 19345217 10.1016/j.febslet.2009.03.065

[65] Rustom A, Saffrich R, Markovic I, Walther P, Gerdes HH. Nanotubular highways for intercellular organelle transport. Science. 2004;303(5660):1007–10. 14963329 10.1126/science.1093133

[66] He K, Shi X, Zhang X, Dang S, Ma X, Liu F, et al. Long-distance intercellular connectivity between cardiomyocytes and cardiofibroblasts mediated by membrane nanotubes. Cardiovasc Res. 2011;92(1):39–47. 21719573 10.1093/cvr/cvr189

[67] Liu K, Ji K, Guo L, Wu W, Lu H, Shan P, et al. Mesenchymal stem cells rescue injured endothelial cells in an in vitro ischemia–reperfusion model via tunneling nanotube like structure-mediated mitochondrial transfer. Microvasc Res. 2014;92:10–8. 24486322 10.1016/j.mvr.2014.01.008

[68] Han H, Hu J, Yan Q, Zhu J, Zhu Z, Chen Y, et al. Bone marrow-derived mesenchymal stem cells rescue injured H9c2 cells via transferring intact mitochondria through tunneling nanotubes in an in vitro simulated ischemia/reperfusion model. Mol Med Rep. 2016;13(2):1517–24. 26718099 10.3892/mmr.2015.4726 PMC4732861

[69] Jackson MV, Morrison TJ, Doherty DF, McAuley DF, Matthay MA, Kissenpfennig A, et al. Mitochondrial transfer via tunneling nanotubes is an important mechanism by which mesenchymal stem cells enhance macrophage phagocytosis in the in vitro and in vivo models of ARDS. Stem Cells. 2016;34(8):2210–23. 27059413 10.1002/stem.2372 PMC4982045

[70] Wang Y, Cui J, Sun X, Zhang Y. Tunneling-nanotube development in astrocytes depends on p53 activation. Cell Death Differ. 2011;18(4):732–42. 21113142 10.1038/cdd.2010.147 PMC3131904

[71] Lou E, Fujisawa S, Morozov A, Barlas A, Romin Y, Dogan Y, et al. Tunneling nanotubes provide a unique conduit for intercellular transfer of cellular contents in human malignant pleural mesothelioma. PLoS ONE. 2012;7(3): e33093. 22427958 10.1371/journal.pone.0033093 PMC3302868

[72] Pitt JM, Kroemer G, Zitvogel L. Extracellular vesicles: masters of intercellular communication and potential clinical interventions. J Clin Investig. 2016;126(4):1139–43. 27035805 10.1172/JCI87316 PMC4811136

[73] Phinney DG, Di Giuseppe M, Njah J, Sala E, Shiva S, St Croix CM, et al. Mesenchymal stem cells use extracellular vesicles to outsource mitophagy and shuttle microRNAs. Nat Commun. 2015;6:8472. 26442449 10.1038/ncomms9472 PMC4598952

[74] Alvarez-Dolado M, Pardal R, Garcia-Verdugo JM, Fike JR, Lee HO, Pfeffer K, et al. Fusion of bone-marrow-derived cells with Purkinje neurons, cardiomyocytes and hepatocytes. Nature. 2003;425(6961):968–73. 14555960 10.1038/nature02069

[75] Nygren JM, Liuba K, Breitbach M, Stott S, Thorén L, Roell W, et al. Myeloid and lymphoid contribution to non-haematopoietic lineages through irradiation-induced heterotypic cell fusion. Nat Cell Biol. 2008;10(5):584–92. 18425115 10.1038/ncb1721

[76] Oh H, Bradfute SB, Gallardo TD, Nakamura T, Gaussin V, Mishina Y, et al. Cardiac progenitor cells from adult myocardium: homing, differentiation, and fusion after infarction. Proc Natl Acad Sci USA. 2003;100(21):12313–8. 14530411 10.1073/pnas.2132126100 PMC218755

[77] Figeac F, Lesault PF, Le Coz O, Damy T, Souktani R, Trébeau C, et al. Nanotubular crosstalk with distressed cardiomyocytes stimulates the paracrine repair function of mesenchymal stem cells. Stem Cells. 2014;32(1):216–30. 24115309 10.1002/stem.1560

[78] Newell C, Sabouny R, Hittel DS, Shutt TE, Khan A, Klein MS, et al. Mesenchymal stem cells shift mitochondrial dynamics and enhance oxidative phosphorylation in recipient cells. Front Physiol. 2018;9:1572. 30555336 10.3389/fphys.2018.01572 PMC6282049

[79] Islam MN, Das SR, Emin MT, Wei M, Sun L, Westphalen K, et al. Mitochondrial transfer from bone-marrow-derived stromal cells to pulmonary alveoli protects against acute lung injury. Nat Med. 2012;18(5):759–65. 22504485 10.1038/nm.2736 PMC3727429

[80] Berridge MV, Schneider RT, McConnell MJ. Mitochondrial transfer from astrocytes to neurons following ischemic insult: guilt by association? Cell Metab. 2016;24(3):376–8. 27626198 10.1016/j.cmet.2016.08.023

[81] Lyamzaev KG, Nepryakhina OK, Saprunova VB, Bakeeva LE, Pletjushkina OY, Chernyak BV, et al. Novel mechanism of elimination of malfunctioning mitochondria (mitoptosis): formation of mitoptotic bodies and extrusion of mitochondrial material from the cell. Biochem Biophys Acta. 2008;1777(7–8):817–25. 18433711 10.1016/j.bbabio.2008.03.027

[82] Boudreau LH, Duchez AC, Cloutier N, Soulet D, Martin N, Bollinger J, et al. Platelets release mitochondria serving as substrate for bactericidal group IIA-secreted phospholipase A2 to promote inflammation. Blood. 2014;124(14):2173–83. 25082876 10.1182/blood-2014-05-573543 PMC4260364

[83] Nakajima A, Kurihara H, Yagita H, Okumura K, Nakano H. Mitochondrial extrusion through the cytoplasmic vacuoles during cell death. J Biol Chem. 2008;283(35):24128–35. 18593703 10.1074/jbc.M802996200 PMC3259776

[84] Popov LD. Mitochondrial biogenesis: an update. J Cell Mol Med. 2020;24(9):4892–9. 32279443 10.1111/jcmm.15194 PMC7205802

[85] Cardanho-Ramos C, Morais VA. Mitochondrial biogenesis in neurons: how and where. Int J Mol Sci. 2021;22(23):13059. 34884861 10.3390/ijms222313059 PMC8657637

[86] Quirós PM, Mottis A, Auwerx J. Mitonuclear communication in homeostasis and stress. Nat Rev Mol Cell Biol. 2016;17(4):213–26. 26956194 10.1038/nrm.2016.23

[87] Baird L, Yamamoto M. The molecular mechanisms regulating the KEAP1–NRF2 pathway. Mol Cell Biol. 2020;40(13): e00099-20. 32284348 10.1128/MCB.00099-20 PMC7296212

[88] Li S, Wen P, Zhang D, Li D, Gao Q, Liu H, et al. PGAM5 expression levels in heart failure and protection ROS-induced oxidative stress and ferroptosis by Keap1/Nrf2. Clin Exp Hypertens. 2023;45(1):2162537. 36780919 10.1080/10641963.2022.2162537

[89] Huang CY, Deng JS, Huang WC, Jiang WP, Huang GJ. Attenuation of lipopolysaccharide-induced acute lung injury by hispolon in mice, through regulating the TLR4/PI3K/Akt/mTOR and Keap1/Nrf2/HO-1 pathways, and suppressing oxidative stress-mediated ER stress-induced apoptosis and autophagy. Nutrients. 2020;12(6):1742. 32532087 10.3390/nu12061742 PMC7352175

[90] Li X, Li J, Zhu D, Zhang N, Hao X, Zhang W, et al. Protein disulfide isomerase PDI-6 regulates Wnt secretion to coordinate inter-tissue UPR(mt) activation and lifespan extension in *C. elegans*. Cell Rep. 2022;39(10): 110931. 35675782 10.1016/j.celrep.2022.110931

[91] Maldonado E, Rojas DA, Urbina F, Solari A. The use of antioxidants as potential co-adjuvants to treat chronic Chagas disease. Antioxidants. 2021;10(7):1022. 34202043 10.3390/antiox10071022 PMC8300663

[92] Chong H, Wei Z, Na M, Sun G, Zheng S, Zhu X, et al. The PGC-1α/NRF1/miR-378a axis protects vascular smooth muscle cells from FFA-induced proliferation, migration and inflammation in atherosclerosis. Atherosclerosis. 2020;297:136–45. 32120345 10.1016/j.atherosclerosis.2020.02.001

[93] Peng T, Xie Y, Sheng H, Wang C, Lian Y, Xie N. Mitochondrial-derived vesicles: gatekeepers of mitochondrial response to oxidative stress. Free Radic Biol Med. 2022;188:185–93. 35750270 10.1016/j.freeradbiomed.2022.06.233

[94] Wu Y, Sun L, Zhuang Z, Hu X, Dong D. Mitochondrial-derived peptides in diabetes and its complications. Front Endocrinol. 2021;12: 808120. 10.3389/fendo.2021.808120 PMC8851315 35185787

[95] Merry TL, Chan A, Woodhead JST, Reynolds JC, Kumagai H, Kim SJ, et al. Mitochondrial-derived peptides in energy metabolism. Am J Physiol Endocrinol Metab. 2020;319(4):E659–66. 32776825 10.1152/ajpendo.00249.2020 PMC7750512

[96] Kim SJ, Miller B, Kumagai H, Silverstein AR, Flores M, Yen K. Mitochondrial-derived peptides in aging and age-related diseases. GeroScience. 2021;43(3):1113–21. 32910336 10.1007/s11357-020-00262-5 PMC8190245

[97] Niikura T, Chiba T, Aiso S, Matsuoka M, Nishimoto I. Humanin: after the discovery. Mol Neurobiol. 2004;30(3):327–40. 15655255 10.1385/MN:30:3:327

[98] Gilon C, Gitlin-Domagalska A, Lahiani A, Yehoshua-Alshanski S, Shumacher-Klinger A, Gilon D, et al. Novel humanin analogs confer neuroprotection and myoprotection to neuronal and myoblast cell cultures exposed to ischemia-like and doxorubicin-induced cell death insults. Peptides. 2020;134: 170399. 32889021 10.1016/j.peptides.2020.170399

[99] Boutari C, Pappas PD, Theodoridis TD, Vavilis D. Humanin and diabetes mellitus: a review of in vitro and in vivo studies. World J Diabetes. 2022;13(3):213–23. 35432758 10.4239/wjd.v13.i3.213 PMC8984571

[100] Rochette L, Meloux A, Zeller M, Cottin Y, Vergely C. Role of humanin, a mitochondrial-derived peptide, in cardiovascular disorders. Arch Cardiovasc Dis. 2020;113(8–9):564–71. 32680738 10.1016/j.acvd.2020.03.020

[101] Gong Y, Luo Y, Liu S, Ma J, Liu F, Fang Y, et al. Pentacyclic triterpene oleanolic acid protects against cardiac aging through regulation of mitophagy and mitochondrial integrity. Biochim Biophys Acta. 2022;1868(7): 166402. 10.1016/j.bbadis.2022.166402 35346820

[102] Hazafa A, Batool A, Ahmad S, Amjad M, Chaudhry SN, Asad J, et al. Humanin: a mitochondrial-derived peptide in the treatment of apoptosis-related diseases. Life Sci. 2021;264: 118679. 33130077 10.1016/j.lfs.2020.118679

[103] Coradduzza D, Congiargiu A, Chen Z, Cruciani S, Zinellu A, Carru C, et al. Humanin and its pathophysiological roles in aging: a systematic review. Biology. 2023;12(4):558. 37106758 10.3390/biology12040558 PMC10135985

[104] Wijenayake S, Storey KB. The role of humanin in natural stress tolerance: an underexplored therapeutic avenue. Biochim Biophys Acta. 2022;1866(1): 130022. 10.1016/j.bbagen.2021.130022 34626747

[105] Xiao J, Kim SJ, Cohen P, Yen K. Humanin: functional interfaces with IGF-I. Growth Horm IGF Res. 2016;29:21–7. 27082450 10.1016/j.ghir.2016.03.005 PMC4961574

[106] Lee C, Zeng J, Drew BG, Sallam T, Martin-Montalvo A, Wan J, et al. The mitochondrial-derived peptide MOTS-c promotes metabolic homeostasis and reduces obesity and insulin resistance. Cell Metab. 2015;21(3):443–54. 25738459 10.1016/j.cmet.2015.02.009 PMC4350682

[107] Zhang Y, Yin K, Wang D, Wang Y, Lu H, Zhao H, et al. Polystyrene microplastics-induced cardiotoxicity in chickens via the ROS-driven NF-κB-NLRP3-GSDMD and AMPK-PGC-1α axes. Sci Total Environ. 2022;840: 156727. 35714743 10.1016/j.scitotenv.2022.156727

[108] Guo Y, Guan T, Shafiq K, Yu Q, Jiao X, Na D, et al. Mitochondrial dysfunction in aging. Ageing Res Rev. 2023;88: 101955. 37196864 10.1016/j.arr.2023.101955

[109] Benayoun BA, Lee C. MOTS-c: a mitochondrial-encoded regulator of the nucleus. BioEssays News Rev Mol Cell Dev Biol. 2019;41(9): e1900046. 10.1002/bies.201900046 PMC8224472 31378979

[110] Gao Y, Wei X, Wei P, Lu H, Zhong L, Tan J, et al. MOTS-c functionally prevents metabolic disorders. Metabolites. 2023;13(1):125. 36677050 10.3390/metabo13010125 PMC9866798

[111] Cobb LJ, Lee C, Xiao J, Yen K, Wong RG, Nakamura HK, et al. Naturally occurring mitochondrial-derived peptides are age-dependent regulators of apoptosis, insulin sensitivity, and inflammatory markers. Aging. 2016;8(4):796–809. 27070352 10.18632/aging.100943 PMC4925829

[112] Mendelsohn AR, Larrick JW. Mitochondrial-derived peptides exacerbate senescence. Rejuvenation Res. 2018;21(4):369–73. 30058454 10.1089/rej.2018.2114

[113] Gong Z, Tas E, Muzumdar R. Humanin and age-related diseases: a new link? Front Endocrinol. 2014;5:210. 10.3389/fendo.2014.00210 PMC4255622 25538685

[114] Miller B, Kim SJ, Kumagai H, Yen K, Cohen P. Mitochondria-derived peptides in aging and healthspan. J Clin Investig. 2022. 10.1172/JCI158449. 35499074 10.1172/JCI158449 PMC9057581

[115] Özcan S, Alessio N, Acar MB, Mert E, Omerli F, Peluso G, et al. Unbiased analysis of senescence associated secretory phenotype (SASP) to identify common components following different genotoxic stresses. Aging. 2016;8(7):1316–29. 27288264 10.18632/aging.100971 PMC4993333

[116] Sreekumar PG, Ishikawa K, Spee C, Mehta HH, Wan J, Yen K, et al. The mitochondrial-derived peptide humanin protects RPE cells from oxidative stress, senescence, and mitochondrial dysfunction. Invest Ophthalmol Vis Sci. 2016;57(3):1238–53. 26990160 10.1167/iovs.15-17053 PMC4811181

[117] van Niel G, D’Angelo G, Raposo G. Shedding light on the cell biology of extracellular vesicles. Nat Rev Mol Cell Biol. 2018;19(4):213–28. 29339798 10.1038/nrm.2017.125

[118] Jeppesen DK, Zhang Q, Franklin JL, Coffey RJ. Extracellular vesicles and nanoparticles: emerging complexities. Trends Cell Biol. 2023;33(8):667–81. 36737375 10.1016/j.tcb.2023.01.002 PMC10363204

[119] Zhou X, Liu S, Lu Y, Wan M, Cheng J, Liu J. MitoEVs: a new player in multiple disease pathology and treatment. J Extracell Vesicles. 2023;12(4): e12320. 37002588 10.1002/jev2.12320 PMC10065981

[120] König T, McBride HM. Mitochondrial-derived vesicles in metabolism, disease, and aging. Cell Metab. 2024;36(1):21–35. 38171335 10.1016/j.cmet.2023.11.014

[121] König T, Nolte H, Aaltonen MJ, Tatsuta T, Krols M, Stroh T, et al. MIROs and DRP1 drive mitochondrial-derived vesicle biogenesis and promote quality control. Nat Cell Biol. 2021;23(12):1271–86. 34873283 10.1038/s41556-021-00798-4

[122] Cadete VJ, Deschênes S, Cuillerier A, Brisebois F, Sugiura A, Vincent A, et al. Formation of mitochondrial-derived vesicles is an active and physiologically relevant mitochondrial quality control process in the cardiac system. J Physiol. 2016;594(18):5343–62. 27311616 10.1113/JP272703 PMC5023710

[123] McLelland GL, Soubannier V, Chen CX, McBride HM, Fon EA. Parkin and PINK1 function in a vesicular trafficking pathway regulating mitochondrial quality control. EMBO J. 2014;33(4):282–95. 24446486 10.1002/embj.201385902 PMC3989637

[124] Howard M, Erickson J, Cuba Z, Kim S, Zhou W, Gade P, et al. A secretory form of Parkin-independent mitophagy contributes to the repertoire of extracellular vesicles released into the tumour interstitial fluid in vivo. J Extracell Vesicles. 2022;11(7): e12244. 35879267 10.1002/jev2.12244 PMC9314315

[125] Lee-Glover LP, Shutt TE. Mitochondrial quality control pathways sense mitochondrial protein import. Trends Endocrinol Metab. 2024;35(4):308–20. 38103974 10.1016/j.tem.2023.11.004

[126] Sugiura A, McLelland GL, Fon EA, McBride HM. A new pathway for mitochondrial quality control: mitochondrial-derived vesicles. EMBO J. 2014;33(19):2142–56. 25107473 10.15252/embj.201488104 PMC4282503

[127] Mondal P, Towers C. Beyond mitophagy: mitochondrial-derived vesicles can get the job done! Autophagy. 2022;18(2):449–51. 34781816 10.1080/15548627.2021.1999562 PMC8942527

[128] Popov LD. Mitochondrial-derived vesicles: recent insights. J Cell Mol Med. 2022;26(12):3323–8. 35582908 10.1111/jcmm.17391 PMC9189329

[129] Soubannier V, McLelland GL, Zunino R, Braschi E, Rippstein P, Fon EA, et al. A vesicular transport pathway shuttles cargo from mitochondria to lysosomes. Curr Biol. 2012;22(2):135–41. 22226745 10.1016/j.cub.2011.11.057

[130] Picca A, Guerra F, Calvani R, Coelho-Junior HJ, Bossola M, Landi F, et al. Generation and release of mitochondrial-derived vesicles in health, aging and disease. J Clin Med. 2020;9(5):1440. 32408624 10.3390/jcm9051440 PMC7290979

[131] Picca A, Guerra F, Calvani R, Coelho-Júnior HJ, Landi F, Bucci C, et al. Mitochondrial-derived vesicles: the good, the bad, and the ugly. Int J Mol Sci. 2023;24(18):13835. 37762138 10.3390/ijms241813835 PMC10531235

[132] Roberts RF, Tang MY, Fon EA, Durcan TM. Defending the mitochondria: the pathways of mitophagy and mitochondrial-derived vesicles. Int J Biochem Cell Biol. 2016;79:427–36. 27443527 10.1016/j.biocel.2016.07.020

[133] Todkar K, Chikhi L, Desjardins V, El-Mortada F, Pépin G, Germain M. Selective packaging of mitochondrial proteins into extracellular vesicles prevents the release of mitochondrial DAMPs. Nat Commun. 2021;12(1):1971. 33785738 10.1038/s41467-021-21984-w PMC8009912

[134] Takenaga K, Koshikawa N, Nagase H. Intercellular transfer of mitochondrial DNA carrying metastasis-enhancing pathogenic mutations from high- to low-metastatic tumor cells and stromal cells via extracellular vesicles. BMC Mol Cell Biol. 2021;22(1):52. 34615464 10.1186/s12860-021-00391-5 PMC8496074

[135] Lazo S, Noren Hooten N, Green J, Eitan E, Mode NA, Liu QR, et al. Mitochondrial DNA in extracellular vesicles declines with age. Aging Cell. 2021;20(1): e13283. 33355987 10.1111/acel.13283 PMC7811845

[136] McLelland GL, Lee SA, McBride HM, Fon EA. Syntaxin-17 delivers PINK1/parkin-dependent mitochondrial vesicles to the endolysosomal system. J Cell Biol. 2016;214(3):275–91. 27458136 10.1083/jcb.201603105 PMC4970327

[137] Vasam G, Nadeau R, Cadete VJJ, Lavallée-Adam M, Menzies KJ, Burelle Y. Proteomics characterization of mitochondrial-derived vesicles under oxidative stress. FASEB J. 2021;35(4): e21278. 33769614 10.1096/fj.202002151R PMC8252493

[138] Ryan TA, Phillips EO, Collier CL, Jb Robinson A, Routledge D, Wood RE, et al. Tollip coordinates Parkin-dependent trafficking of mitochondrial-derived vesicles. EMBO J. 2020;39(11): e102539. 32311122 10.15252/embj.2019102539 PMC7265236

[139] Terešak P, Lapao A, Subic N, Boya P, Elazar Z, Simonsen A. Regulation of PRKN-independent mitophagy. Autophagy. 2022;18(1):24–39. 33570005 10.1080/15548627.2021.1888244 PMC8865282

[140] Roberts RF, Fon EA. Presenting mitochondrial antigens: PINK1, Parkin and MDVs steal the show. Cell Res. 2016;26(11):1180–1. 27585536 10.1038/cr.2016.104 PMC5099861

[141] Manickam DS. Delivery of mitochondria via extracellular vesicles—a new horizon in drug delivery. J Control Release. 2022;343:400–7. 35131369 10.1016/j.jconrel.2022.01.045

[142] Velarde F, Ezquerra S, Delbruyere X, Caicedo A, Hidalgo Y, Khoury M. Mesenchymal stem cell-mediated transfer of mitochondria: mechanisms and functional impact. Cell Mol Life Sci. 2022;79(3):177. 35247083 10.1007/s00018-022-04207-3 PMC11073024

[143] Sanz-Ros J, Mas-Bargues C, Romero-García N, Huete-Acevedo J, Dromant M, Borrás C. The potential use of mitochondrial extracellular vesicles as biomarkers or therapeutical tools. Int J Mol Sci. 2023;24(8):7005. 37108168 10.3390/ijms24087005 PMC10139054

[144] Amari L, Germain M. Mitochondrial extracellular vesicles—origins and roles. Front Mol Neurosci. 2021;14: 767219. 34751216 10.3389/fnmol.2021.767219 PMC8572053

[145] Roberts RF, Bayne AN, Goiran T, Lévesque D, Boisvert FM, Trempe JF, et al. Proteomic profiling of mitochondrial-derived vesicles in brain reveals enrichment of respiratory complex sub-assemblies and small TIM chaperones. J Proteome Res. 2021;20(1):506–17. 33242952 10.1021/acs.jproteome.0c00506

[146] Mohanty A, Zunino R, Soubannier V, Dilipkumar S. A new functional role of mitochondria-anchored protein ligase in peroxisome morphology in mammalian cells. J Cell Biochem. 2021;122(11):1686–700. 34322908 10.1002/jcb.30114

[147] Di Florio DN, Beetler DJ, McCabe EJ, Sin J, Ikezu T, Fairweather D. Mitochondrial extracellular vesicles, autoimmunity and myocarditis. Front Immunol. 2024;15:1374796. 38550582 10.3389/fimmu.2024.1374796 PMC10972887

[148] Ryan TA, Tumbarello DA. A central role for mitochondrial-derived vesicles in the innate immune response: implications for Parkinson’s disease. Neural Regen Res. 2021;16(9):1779–80. 33510074 10.4103/1673-5374.306074 PMC8328781

[149] Rosina M, Ceci V, Turchi R, Chuan L, Borcherding N, Sciarretta F, et al. Ejection of damaged mitochondria and their removal by macrophages ensure efficient thermogenesis in brown adipose tissue. Cell Metab. 2022;34(4):533-48.e12. 35305295 10.1016/j.cmet.2022.02.016 PMC9039922

[150] Ikeda G, Santoso MR, Tada Y, Li AM, Vaskova E, Jung JH, et al. Mitochondria-rich extracellular vesicles from autologous stem cell-derived cardiomyocytes restore energetics of ischemic myocardium. J Am Coll Cardiol. 2021;77(8):1073–88. 33632482 10.1016/j.jacc.2020.12.060 PMC8626617

[151] Towers CG, Wodetzki DK, Thorburn J, Smith KR, Caino MC, Thorburn A. Mitochondrial-derived vesicles compensate for loss of LC3-mediated mitophagy. Dev Cell. 2021;56(14):2029-42.e5. 34171288 10.1016/j.devcel.2021.06.003 PMC8319140

[152] Ramirez A, Old W, Selwood DL, Liu X. Cannabidiol activates PINK1-Parkin-dependent mitophagy and mitochondrial-derived vesicles. Eur J Cell Biol. 2022;101(1): 151185. 34915361 10.1016/j.ejcb.2021.151185 PMC8816654

[153] Chaiyarit S, Thongboonkerd V. Mitochondria-derived vesicles and their potential roles in kidney stone disease. J Transl Med. 2023;21(1):294. 37131163 10.1186/s12967-023-04133-3 PMC10152607

[154] Chandel NS. Evolution of mitochondria as signaling organelles. Cell Metab. 2015;22(2):204–6. 26073494 10.1016/j.cmet.2015.05.013

[155] Frezza C. Mitochondrial metabolites: undercover signalling molecules. Interface focus. 2017;7(2):20160100. 28382199 10.1098/rsfs.2016.0100 PMC5311903

[156] Allis CD, Jenuwein T. The molecular hallmarks of epigenetic control. Nat Rev Genet. 2016;17(8):487–500. 27346641 10.1038/nrg.2016.59

[157] Shaughnessy DT, McAllister K, Worth L, Haugen AC, Meyer JN, Domann FE, et al. Mitochondria, energetics, epigenetics, and cellular responses to stress. Environ Health Perspect. 2014;122(12):1271–8. 25127496 10.1289/ehp.1408418 PMC4256704

[158] Fetterman JL, Ballinger SW. Mitochondrial genetics regulate nuclear gene expression through metabolites. Proc Natl Acad Sci USA. 2019;116(32):15763–5. 31308238 10.1073/pnas.1909996116 PMC6689900

[159] Feeley KP, Bray AW, Westbrook DG, Johnson LW, Kesterson RA, Ballinger SW, et al. Mitochondrial genetics regulate breast cancer tumorigenicity and metastatic potential. Can Res. 2015;75(20):4429–36. 10.1158/0008-5472.CAN-15-0074 PMC4610037 26471915

[160] Sagar S, Kapoor H, Chaudhary N, Roy SS. Cellular and mitochondrial calcium communication in obstructive lung disorders. Mitochondrion. 2021;58:184–99. 33766748 10.1016/j.mito.2021.03.005

[161] Yousuf MS, Maguire AD, Simmen T, Kerr BJ. Endoplasmic reticulum-mitochondria interplay in chronic pain: the calcium connection. Mol Pain. 2020;16:1744806920946889. 32787562 10.1177/1744806920946889 PMC7427143

[162] Rizzuto R, De Stefani D, Raffaello A, Mammucari C. Mitochondria as sensors and regulators of calcium signalling. Nat Rev Mol Cell Biol. 2012;13(9):566–78. 22850819 10.1038/nrm3412

[163] Kaufman RJ, Malhotra JD. Calcium trafficking integrates endoplasmic reticulum function with mitochondrial bioenergetics. Biochem Biophys Acta. 2014;1843(10):2233–9. 24690484 10.1016/j.bbamcr.2014.03.022 PMC4285153

[164] Alevriadou BR, Patel A, Noble M, Ghosh S, Gohil VM, Stathopulos PB, et al. Molecular nature and physiological role of the mitochondrial calcium uniporter channel. Am J Physiol Cell Physiol. 2021;320(4):C465–82. 33296287 10.1152/ajpcell.00502.2020 PMC8260355

[165] Cortassa S, O’Rourke B, Winslow RL, Aon MA. Control and regulation of integrated mitochondrial function in metabolic and transport networks. Int J Mol Sci. 2009;10(4):1500–13. 19468321 10.3390/ijms10041500 PMC2680629

[166] Liu T, O’Rourke B. Regulation of mitochondrial Ca2+ and its effects on energetics and redox balance in normal and failing heart. J Bioenerg Biomembr. 2009;41(2):127–32. 19390955 10.1007/s10863-009-9216-8 PMC2946065

[167] Weidinger A, Milivojev N, Hosmann A, Duvigneau JC, Szabo C, Törö G, et al. Oxoglutarate dehydrogenase complex controls glutamate-mediated neuronal death. Redox Biol. 2023;62: 102669. 36933393 10.1016/j.redox.2023.102669 PMC10031542

[168] Pitt D, Mosley MJ. Oxidation of carbon sources via the tricarboxylic acid cycle during calcium-induced conidiation of *Penicillium notatum*. Antonie Van Leeuwenhoek. 1986;52(6):467–82. 3813521 10.1007/BF00423408

[169] Xian H, Watari K, Sanchez-Lopez E, Offenberger J, Onyuru J, Sampath H, et al. Oxidized DNA fragments exit mitochondria via mPTP- and VDAC-dependent channels to activate NLRP3 inflammasome and interferon signaling. Immunity. 2022;55(8):1370-85.e8. 35835107 10.1016/j.immuni.2022.06.007 PMC9378606

[170] De Gaetano A, Solodka K, Zanini G, Selleri V, Mattioli AV, Nasi M, et al. Molecular mechanisms of mtDNA-mediated inflammation. Cells. 2021;10(11):2898. 34831121 10.3390/cells10112898 PMC8616383

[171] Jiménez-Loygorri JI, Villarejo-Zori B, Viedma-Poyatos Á, Zapata-Muñoz J, Benítez-Fernández R, Frutos-Lisón MD, et al. Mitophagy curtails cytosolic mtDNA-dependent activation of cGAS/STING inflammation during aging. Nat Commun. 2024;15(1):830. 38280852 10.1038/s41467-024-45044-1 PMC10821893

[172] Decout A, Katz JD, Venkatraman S, Ablasser A. The cGAS-STING pathway as a therapeutic target in inflammatory diseases. Nat Rev Immunol. 2021;21(9):548–69. 33833439 10.1038/s41577-021-00524-z PMC8029610

[173] Oduro PK, Zheng X, Wei J, Yang Y, Wang Y, Zhang H, et al. The cGAS-STING signaling in cardiovascular and metabolic diseases: future novel target option for pharmacotherapy. Acta Pharm Sin B. 2022;12(1):50–75. 35127372 10.1016/j.apsb.2021.05.011 PMC8799861

[174] Kim J, Kim HS, Chung JH. Molecular mechanisms of mitochondrial DNA release and activation of the cGAS-STING pathway. Exp Mol Med. 2023;55(3):510–9. 36964253 10.1038/s12276-023-00965-7 PMC10037406

[175] López-Otín C, Blasco MA, Partridge L, Serrano M, Kroemer G. Hallmarks of aging: an expanding universe. Cell. 2023;186(2):243–78. 36599349 10.1016/j.cell.2022.11.001

[176] Miwa S, Kashyap S, Chini E, von Zglinicki T. Mitochondrial dysfunction in cell senescence and aging. J Clin Investig. 2022. 10.1172/JCI158447. 35775483 10.1172/JCI158447 PMC9246372

[177] Tian R, Colucci WS, Arany Z, Bachschmid MM, Ballinger SW, Boudina S, et al. Unlocking the secrets of mitochondria in the cardiovascular system: path to a cure in heart failure—a report from the 2018 national heart, lung, and blood institute workshop. Circulation. 2019;140(14):1205–16. 31769940 10.1161/CIRCULATIONAHA.119.040551 PMC6880654

[178] Song J, Herrmann JM, Becker T. Quality control of the mitochondrial proteome. Nat Rev Mol Cell Biol. 2021;22(1):54–70. 33093673 10.1038/s41580-020-00300-2

[179] Wu L, Wang L, Du Y, Zhang Y, Ren J. Mitochondrial quality control mechanisms as therapeutic targets in doxorubicin-induced cardiotoxicity. Trends Pharmacol Sci. 2023;44(1):34–49. 36396497 10.1016/j.tips.2022.10.003

[180] Keerthiga R, Pei DS, Fu A. Mitochondrial dysfunction, UPR(mt) signaling, and targeted therapy in metastasis tumor. Cell Biosci. 2021;11(1):186. 34717757 10.1186/s13578-021-00696-0 PMC8556915

[181] Ducker GS, Rabinowitz JD. One-carbon metabolism in health and disease. Cell Metab. 2017;25(1):27–42. 27641100 10.1016/j.cmet.2016.08.009 PMC5353360

[182] Reina-Campos M, Diaz-Meco MT, Moscat J. The complexity of the serine glycine one-carbon pathway in cancer. J Cell Biol. 2020;219(1): e201907022. 31690618 10.1083/jcb.201907022 PMC7039202

[183] Castegna A, Iacobazzi V, Infantino V. The mitochondrial side of epigenetics. Physiol Genomics. 2015;47(8):299–307. 26038395 10.1152/physiolgenomics.00096.2014

[184] Agrimi G, Di Noia MA, Marobbio CM, Fiermonte G, Lasorsa FM, Palmieri F. Identification of the human mitochondrial S-adenosylmethionine transporter: bacterial expression, reconstitution, functional characterization and tissue distribution. Biochem J. 2004;379(Pt 1):183–90. 14674884 10.1042/BJ20031664 PMC1224042

[185] Gusic M, Prokisch H. ncRNAs: new players in mitochondrial health and disease? Front Genet. 2020;11:95. 32180794 10.3389/fgene.2020.00095 PMC7059738

[186] Calvo-Rodriguez M, Hou SS, Snyder AC, Kharitonova EK, Russ AN, Das S, et al. Increased mitochondrial calcium levels associated with neuronal death in a mouse model of Alzheimer’s disease. Nat Commun. 2020;11(1):2146. 32358564 10.1038/s41467-020-16074-2 PMC7195480

[187] Lung B, Zemann A, Madej MJ, Schuelke M, Techritz S, Ruf S, et al. Identification of small non-coding RNAs from mitochondria and chloroplasts. Nucleic Acids Res. 2006;34(14):3842–52. 16899451 10.1093/nar/gkl448 PMC1557801

[188] Zhang Q, Raoof M, Chen Y, Sumi Y, Sursal T, Junger W, et al. Circulating mitochondrial DAMPs cause inflammatory responses to injury. Nature. 2010;464(7285):104–7. 20203610 10.1038/nature08780 PMC2843437

[189] Nakayama H, Otsu K. Mitochondrial DNA as an inflammatory mediator in cardiovascular diseases. Biochem J. 2018;475(5):839–52. 29511093 10.1042/BCJ20170714 PMC5840331

[190] Wu Z, Oeck S, West AP, Mangalhara KC, Sainz AG, Newman LE, et al. Mitochondrial DNA stress signalling protects the nuclear genome. Nat Metab. 2019;1(12):1209–18. 32395698 10.1038/s42255-019-0150-8 PMC7213273

[191] Qin C, Gu J, Hu J, Qian H, Fei X, Li Y, et al. Platelets activation is associated with elevated plasma mitochondrial DNA during cardiopulmonary bypass. J Cardiothorac Surg. 2016;11(1):90. 27266529 10.1186/s13019-016-0481-4 PMC4895797

[192] Bliksøen M, Mariero LH, Torp MK, Baysa A, Ytrehus K, Haugen F, et al. Extracellular mtDNA activates NF-κB via toll-like receptor 9 and induces cell death in cardiomyocytes. Basic Res Cardiol. 2016;111(4):42. 27164906 10.1007/s00395-016-0553-6

[193] Picca A, Guerra F, Calvani R, Coelho-Júnior HJ, Landi F, Bernabei R, et al. Extracellular vesicles and damage-associated molecular patterns: a Pandora’s box in health and disease. Front Immunol. 2020;11: 601740. 33304353 10.3389/fimmu.2020.601740 PMC7701251

[194] Dong LF, Rohlena J, Zobalova R, Nahacka Z, Rodriguez AM, Berridge MV, et al. Mitochondria on the move: horizontal mitochondrial transfer in disease and health. J Cell Biol. 2023;222(3): e202211044. 36795453 10.1083/jcb.202211044 PMC9960264

[195] Chang JC, Wu SL, Liu KH, Chen YH, Chuang CS, Cheng FC, et al. Allogeneic/xenogeneic transplantation of peptide-labeled mitochondria in Parkinson’s disease: restoration of mitochondria functions and attenuation of 6-hydroxydopamine-induced neurotoxicity. Transl Res. 2016;170:40-56.e3. 26730494 10.1016/j.trsl.2015.12.003

[196] Ahn BH, Kim HS, Song S, Lee IH, Liu J, Vassilopoulos A, et al. A role for the mitochondrial deacetylase Sirt3 in regulating energy homeostasis. Proc Natl Acad Sci USA. 2008;105(38):14447–52. 18794531 10.1073/pnas.0803790105 PMC2567183

[197] Bach D, Pich S, Soriano FX, Vega N, Baumgartner B, Oriola J, et al. Mitofusin-2 determines mitochondrial network architecture and mitochondrial metabolism. A novel regulatory mechanism altered in obesity. J Biol Chem. 2003;278(19):17190–7. 12598526 10.1074/jbc.M212754200

[198] Theurey P, Rieusset J. Mitochondria-associated membranes response to nutrient availability and role in metabolic diseases. Trends Endocrinol Metab. 2017;28(1):32–45. 27670636 10.1016/j.tem.2016.09.002

[199] Andreux PA, Houtkooper RH, Auwerx J. Pharmacological approaches to restore mitochondrial function. Nat Rev Drug Discov. 2013;12(6):465–83. 23666487 10.1038/nrd4023 PMC3896945

[200] Gong B, Pan Y, Vempati P, Zhao W, Knable L, Ho L, et al. Nicotinamide riboside restores cognition through an upregulation of proliferator-activated receptor-γ coactivator 1α regulated β-secretase 1 degradation and mitochondrial gene expression in Alzheimer’s mouse models. Neurobiol Aging. 2013;34(6):1581–8. 23312803 10.1016/j.neurobiolaging.2012.12.005 PMC3632303

[201] Xu Z, Huo J, Ding X, Yang M, Li L, Dai J, et al. Coenzyme Q10 improves lipid metabolism and ameliorates obesity by regulating CaMKII-mediated PDE4 inhibition. Sci Rep. 2017;7(1):8253. 28811612 10.1038/s41598-017-08899-7 PMC5557856

[202] Mohammed I, Hollenberg MD, Ding H, Triggle CR. A critical review of the evidence that metformin is a putative anti-aging drug that enhances healthspan and extends lifespan. Front Endocrinol. 2021;12: 718942. 10.3389/fendo.2021.718942 PMC8374068 34421827

[203] Rodríguez-Bies E, Tung BT, Navas P, López-Lluch G. Resveratrol primes the effects of physical activity in old mice. Br J Nutr. 2016;116(6):979–88. 27488121 10.1017/S0007114516002920

[204] Givvimani S, Munjal C, Tyagi N, Sen U, Metreveli N, Tyagi SC. Mitochondrial division/mitophagy inhibitor (Mdivi) ameliorates pressure overload induced heart failure. PLoS ONE. 2012;7(3): e32388. 22479323 10.1371/journal.pone.0032388 PMC3313999

[205] Rappold PM, Cui M, Grima JC, Fan RZ, de Mesy-Bentley KL, Chen L, et al. Drp1 inhibition attenuates neurotoxicity and dopamine release deficits in vivo. Nat Commun. 2014;5:5244. 25370169 10.1038/ncomms6244 PMC4223875

[206] Joshi AU, Saw NL, Vogel H, Cunnigham AD, Shamloo M, Mochly-Rosen D. Inhibition of Drp1/Fis1 interaction slows progression of amyotrophic lateral sclerosis. EMBO Mol Med. 2018;10(3): e8166. 29335339 10.15252/emmm.201708166 PMC5840540

[207] Disatnik MH, Hwang S, Ferreira JC, Mochly-Rosen D. New therapeutics to modulate mitochondrial dynamics and mitophagy in cardiac diseases. J Mol Med. 2015;93(3):279–87. 25652199 10.1007/s00109-015-1256-4 PMC4333238

[208] Ferreira JC, Mochly-Rosen D. Nitroglycerin use in myocardial infarction patients. Circ J. 2012;76(1):15–21. 22040938 10.1253/circj.cj-11-1133 PMC3527093

[209] Nishimura A, Shimauchi T, Tanaka T, Shimoda K, Toyama T, Kitajima N, et al. Hypoxia-induced interaction of filamin with Drp1 causes mitochondrial hyperfission-associated myocardial senescence. Sci Signal. 2018;11(556): eaat5185. 30425165 10.1126/scisignal.aat5185

[210] Jayatunga DPW, Hone E, Khaira H, Lunelli T, Singh H, Guillemin GJ, et al. Therapeutic potential of mitophagy-inducing microflora metabolite, urolithin A for Alzheimer’s disease. Nutrients. 2021;13(11):3744. 34836000 10.3390/nu13113744 PMC8617978

[211] Qiu J, Chen Y, Zhuo J, Zhang L, Liu J, Wang B, et al. Urolithin A promotes mitophagy and suppresses NLRP3 inflammasome activation in lipopolysaccharide-induced BV2 microglial cells and MPTP-induced Parkinson’s disease model. Neuropharmacology. 2022;207: 108963. 35065082 10.1016/j.neuropharm.2022.108963

[212] Gao Z, Yi W, Tang J, Sun Y, Huang J, Lan T, et al. Urolithin A protects against acetaminophen-induced liver injury in mice via sustained activation of Nrf2. Int J Biol Sci. 2022;18(5):2146–62. 35342347 10.7150/ijbs.69116 PMC8935220

[213] Huang JR, Zhang MH, Chen YJ, Sun YL, Gao ZM, Li ZJ, et al. Urolithin A ameliorates obesity-induced metabolic cardiomyopathy in mice via mitophagy activation. Acta Pharmacol Sin. 2023;44(2):321–31. 35655094 10.1038/s41401-022-00919-1 PMC9889402

[214] Xie C, Zhuang XX, Niu Z, Ai R, Lautrup S, Zheng S, et al. Amelioration of Alzheimer’s disease pathology by mitophagy inducers identified via machine learning and a cross-species workflow. Nat Biomed Eng. 2022;6(1):76–93. 34992270 10.1038/s41551-021-00819-5 PMC8782726

[215] Manczak M, Mao P, Calkins MJ, Cornea A, Reddy AP, Murphy MP, et al. Mitochondria-targeted antioxidants protect against amyloid-beta toxicity in Alzheimer’s disease neurons. J Alzheimer’s Dis. 2010;20(Suppl 2):S609–31. 20463406 10.3233/JAD-2010-100564 PMC3072711

[216] RibeiroJunior RF, Dabkowski ER, Shekar KC, Connell KAO, Hecker PA, Murphy MP. MitoQ improves mitochondrial dysfunction in heart failure induced by pressure overload. Free Radic Biol Med. 2018;117:18–29. 29421236 10.1016/j.freeradbiomed.2018.01.012 PMC5866124

[217] Fink BD, Herlein JA, Guo DF, Kulkarni C, Weidemann BJ, Yu L, et al. A mitochondrial-targeted coenzyme q analog prevents weight gain and ameliorates hepatic dysfunction in high-fat-fed mice. J Pharmacol Exp Ther. 2014;351(3):699–708. 25301169 10.1124/jpet.114.219329 PMC4244581

[218] Reddy PH, Manczak M, Kandimalla R. Mitochondria-targeted small molecule SS31: a potential candidate for the treatment of Alzheimer’s disease. Hum Mol Genet. 2017;26(8):1483–96. 28186562 10.1093/hmg/ddx052 PMC6075532

[219] Dai W, Shi J, Gupta RC, Sabbah HN, Hale SL, Kloner RA. Bendavia, a mitochondria-targeting peptide, improves postinfarction cardiac function, prevents adverse left ventricular remodeling, and restores mitochondria-related gene expression in rats. J Cardiovasc Pharmacol. 2014;64(6):543–53. 25165999 10.1097/FJC.0000000000000155

[220] Shi J, Dai W, Hale SL, Brown DA, Wang M, Han X, et al. Bendavia restores mitochondrial energy metabolism gene expression and suppresses cardiac fibrosis in the border zone of the infarcted heart. Life Sci. 2015;141:170–8. 26431885 10.1016/j.lfs.2015.09.022 PMC4973309

[221] Kamboj SS, Vasishta RK, Sandhir R. *N*-Acetylcysteine inhibits hyperglycemia-induced oxidative stress and apoptosis markers in diabetic neuropathy. J Neurochem. 2010;112(1):77–91. 19840221 10.1111/j.1471-4159.2009.06435.x

[222] Yoshino J, Mills KF, Yoon MJ, Imai S. Nicotinamide mononucleotide, a key NAD(+) intermediate, treats the pathophysiology of diet- and age-induced diabetes in mice. Cell Metab. 2011;14(4):528–36. 21982712 10.1016/j.cmet.2011.08.014 PMC3204926

[223] Karuppagounder SS, Pinto JT, Xu H, Chen HL, Beal MF, Gibson GE. Dietary supplementation with resveratrol reduces plaque pathology in a transgenic model of Alzheimer’s disease. Neurochem Int. 2009;54(2):111–8. 19041676 10.1016/j.neuint.2008.10.008 PMC2892907

[224] Baur JA, Pearson KJ, Price NL, Jamieson HA, Lerin C, Kalra A, et al. Resveratrol improves health and survival of mice on a high-calorie diet. Nature. 2006;444(7117):337–42. 17086191 10.1038/nature05354 PMC4990206

[225] Gérard C, Xiao X, Filali M, Coulombe Z, Arsenault M, Couet J, et al. An AAV9 coding for frataxin clearly improved the symptoms and prolonged the life of Friedreich ataxia mouse models. Mol Ther Methods Clin Dev. 2014;1:14044. 26015982 10.1038/mtm.2014.44 PMC4362356

[226] Rehfeldt SCH, Laufer S, Goettert MI. A highly selective in vitro JNK3 inhibitor, FMU200, restores mitochondrial membrane potential and reduces oxidative stress and apoptosis in SH-SY5Y cells. Int J Mol Sci. 2021;22(7):3701. 33918172 10.3390/ijms22073701 PMC8037381

[227] Han Y, Jiang M, He R, Lv X, Liao X, He Y, et al. Mefunidone ameliorates bleomycin-induced pulmonary fibrosis in mice. Front Pharmacol. 2021;12: 713572. 34630088 10.3389/fphar.2021.713572 PMC8499630

[228] Sorrentino V, Romani M, Mouchiroud L, Beck JS, Zhang H, D’Amico D, et al. Enhancing mitochondrial proteostasis reduces amyloid-β proteotoxicity. Nature. 2017;552(7684):187–93. 29211722 10.1038/nature25143 PMC5730497

[229] Rysted JE, Lin Z, Walters GC, Rauckhorst AJ, Noterman M, Liu G, et al. Distinct properties of Ca(2+) efflux from brain, heart and liver mitochondria: the effects of Na(+), Li(+) and the mitochondrial Na(+)/Ca(2+) exchange inhibitor CGP37157. Cell Calcium. 2021;96: 102382. 33684833 10.1016/j.ceca.2021.102382 PMC8187304

[230] Yoshinaga N, Numata K. Rational designs at the forefront of mitochondria-targeted gene delivery: recent progress and future perspectives. ACS Biomater Sci Eng. 2022;8(2):348–59. 34979085 10.1021/acsbiomaterials.1c01114

[231] Kaza AK, Wamala I, Friehs I, Kuebler JD, Rathod RH, Berra I, et al. Myocardial rescue with autologous mitochondrial transplantation in a porcine model of ischemia/reperfusion. J Thorac Cardiovasc Surg. 2017;153(4):934–43. 27938904 10.1016/j.jtcvs.2016.10.077

[232] Masuzawa A, Black KM, Pacak CA, Ericsson M, Barnett RJ, Drumm C, et al. Transplantation of autologously derived mitochondria protects the heart from ischemia–reperfusion injury. Am J Physiol Heart Circ Physiol. 2013;304(7):H966–82. 23355340 10.1152/ajpheart.00883.2012 PMC3625892

[233] Bertoldo MJ, Listijono DR, Ho WJ, Riepsamen AH, Goss DM, Richani D, et al. NAD(+) repletion rescues female fertility during reproductive aging. Cell Rep. 2020;30(6):1670-81.e7. 32049001 10.1016/j.celrep.2020.01.058 PMC7063679

[234] Chu X, Hou Y, Meng Q, Croteau DL, Wei Y, De S, et al. Nicotinamide adenine dinucleotide supplementation drives gut microbiota variation in Alzheimer’s mouse model. Front Aging Neurosci. 2022;14: 993615. 36185477 10.3389/fnagi.2022.993615 PMC9520302

[235] Ryu D, Mouchiroud L, Andreux PA, Katsyuba E, Moullan N, Nicolet-Dit-Félix AA, et al. Urolithin A induces mitophagy and prolongs lifespan in *C. elegans* and increases muscle function in rodents. Nat Med. 2016;22(8):879–88. 27400265 10.1038/nm.4132

[236] Chavez JD, Tang X, Campbell MD, Reyes G, Kramer PA, Stuppard R, et al. Mitochondrial protein interaction landscape of SS-31. Proc Natl Acad Sci USA. 2020;117(26):15363–73. 32554501 10.1073/pnas.2002250117 PMC7334473

[237] Lagouge M, Argmann C, Gerhart-Hines Z, Meziane H, Lerin C, Daussin F, et al. Resveratrol improves mitochondrial function and protects against metabolic disease by activating SIRT1 and PGC-1alpha. Cell. 2006;127(6):1109–22. 17112576 10.1016/j.cell.2006.11.013

[238] Huang T, Lin R, Su Y, Sun H, Zheng X, Zhang J, et al. Efficient intervention for pulmonary fibrosis via mitochondrial transfer promoted by mitochondrial biogenesis. Nat Commun. 2023;14(1):5781. 37723135 10.1038/s41467-023-41529-7 PMC10507082

[239] Gammage PA, Viscomi C, Simard ML, Costa ASH, Gaude E, Powell CA, et al. Genome editing in mitochondria corrects a pathogenic mtDNA mutation in vivo. Nat Med. 2018;24(11):1691–5. 30250142 10.1038/s41591-018-0165-9 PMC6225988

[240] Zekonyte U, Bacman SR, Smith J, Shoop W, Pereira CV, Tomberlin G, et al. Mitochondrial targeted meganuclease as a platform to eliminate mutant mtDNA in vivo. Nat Commun. 2021;12(1):3210. 34050192 10.1038/s41467-021-23561-7 PMC8163834

[241] Silva-Pinheiro P, Nash PA, Van Haute L, Mutti CD, Turner K, Minczuk M. In vivo mitochondrial base editing via adeno-associated viral delivery to mouse post-mitotic tissue. Nat Commun. 2022;13(1):750. 35136065 10.1038/s41467-022-28358-w PMC8825850

[242] Reichart D, Newby GA, Wakimoto H, Lun M, Gorham JM, Curran JJ, et al. Efficient in vivo genome editing prevents hypertrophic cardiomyopathy in mice. Nat Med. 2023;29(2):412–21. 36797483 10.1038/s41591-022-02190-7 PMC9941048

[243] Kang E, Wu J, Gutierrez NM, Koski A, Tippner-Hedges R, Agaronyan K, et al. Mitochondrial replacement in human oocytes carrying pathogenic mitochondrial DNA mutations. Nature. 2016;540(7632):270–5. 27919073 10.1038/nature20592

